# Supplementary material for: Calibrated simulations for dynamic focusing of ultrasound through the temporal window
Source: Cell Rep Methods. 2026 Aug 14;6(10):101568. doi: 10.1016/j.crmeth.2026.101568 (PMC13721750; doi:10.1016/j.crmeth.2026.101568)
Supplement: Supplementary file 1 [file mmc1.pdf]

**Cell Reports Methods, Volume *6***

## Supplemental information

# Calibrated simulations for dynamic focusing of ultrasound through the temporal window

**Ehsan Dadgar-Kiani, Vibha Hebbale, Malachy W. Newman, Grace Attalla, John L. Alvarez, Suraya Dunsford, Kevin A. Caulfield, Cameron H. Good, Andrew D. Krystal, Leo P. Sugrue, Joline M. Fan, Elsa Fouragnan, Charles F. Caskey, Samuel Pichardo, Kim Butts Pauly, and Keith R. Murphy**

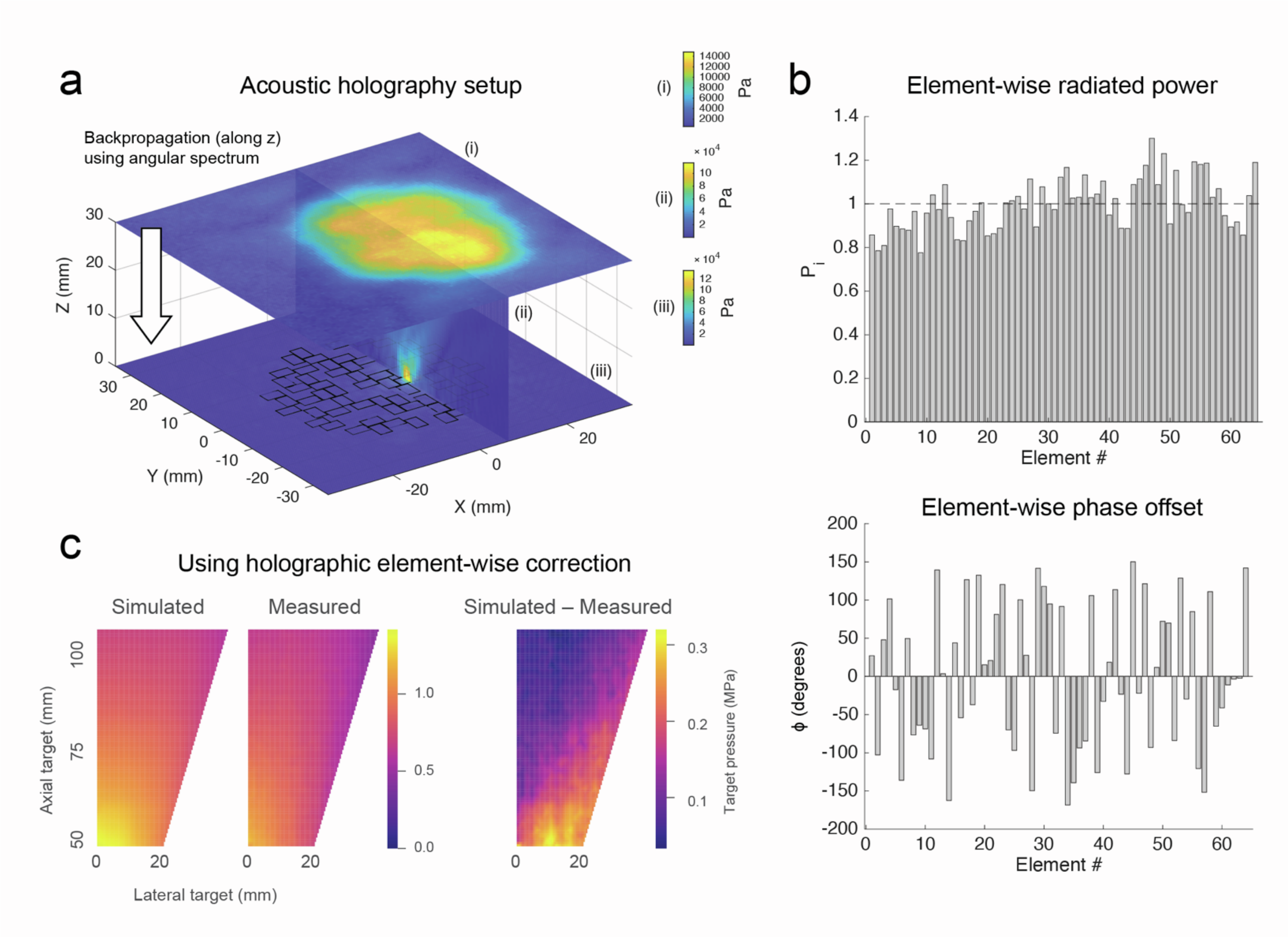


**Figure S1. Holography-based transducer calibration is unable to capture spatially dependent crosstalk when steering a multi-element phased array, Related to Figure 1.** **a**) Depiction of the acoustic holography setup used to calibrate a 2D phased array transducer by measuring the complex acoustic field generated by each element over a two-dimensional plane at a distance of 30 mm from the array plane. This measured complex plane (i) from a single source element is backpropagated using the angular spectrum method to recover the source complex plane (iii). An orthogonal plane (ii) depicts the spatial convergence of the pressure field to the original source element through backpropagation. In (i), (ii), and (iii), only complex magnitude is depicted. **b**) Following backpropagation for each source element, variability in average radiated power and phase offset are observed for each element. **c**) When using the source complex planes to simulate focusing across the steering range, the discrepancy between simulated peak pressures and measured peak pressures still shows a significant spatial dependence, indicating that purely holographic-based transducer calibration is insufficient.

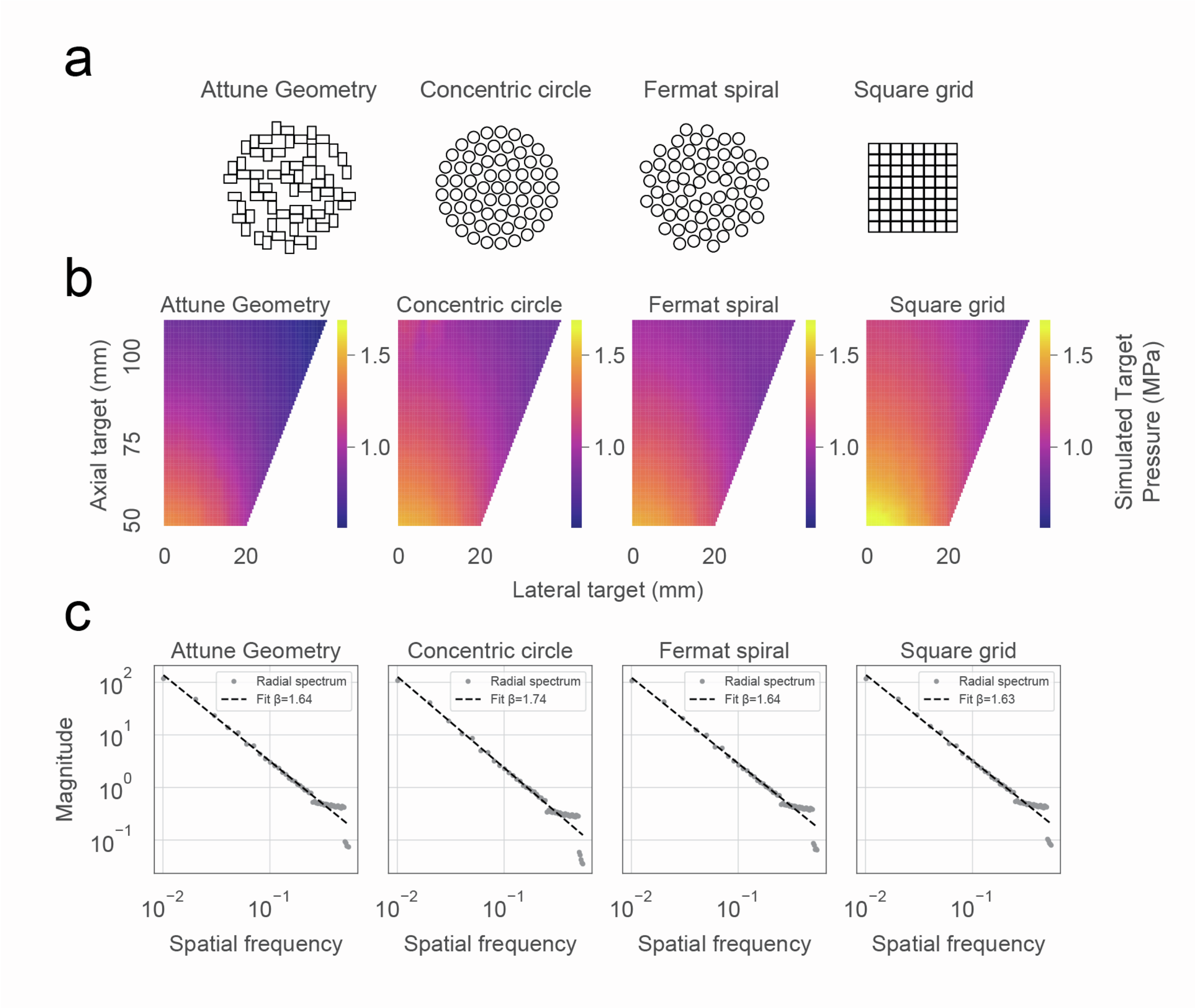


**Figure S2. Variant ultrasound array designs tested for focal pressure output symmetry, Related to Figure 1.** **a**) Depictions of 2D transducer element configurations used to generate **b**) simulated focal pressure maps when steering across a 2D calibration range. **c**) All array configurations' focal pressure maps showed comparable magnitudes across the relevant spatial frequency range.

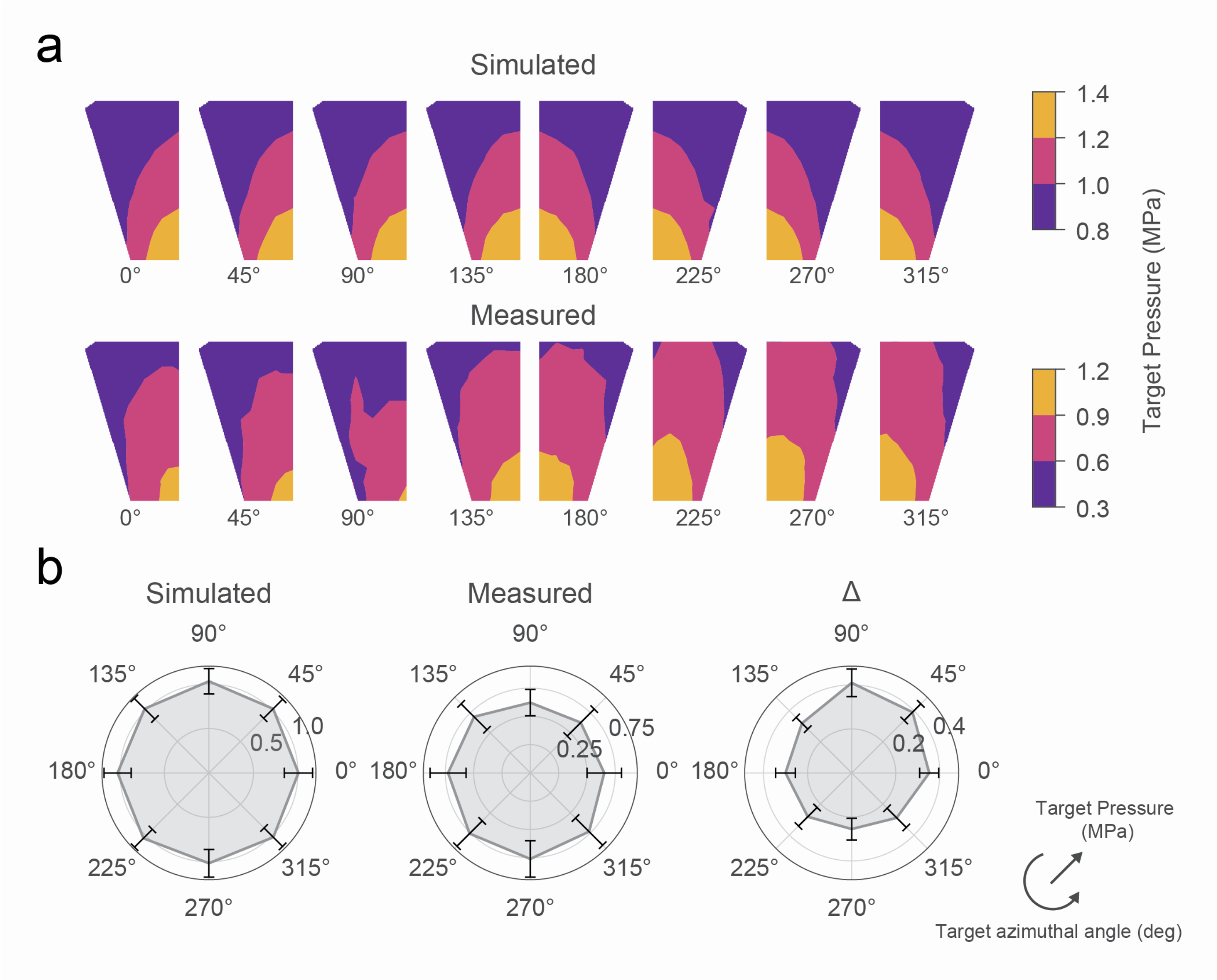


**Figure S3. Focal pressures at variable azimuthal angles show spatial nonuniformity and lateral bias between measurement and simulation, Related to Figure 1. a)** Comparisons between simulated and measured focal pressure cross sections ranging from 0º to 315º in 45º increments. Maps were segmented into three uniform color bins to better depict the uniformity evident in simulation but absent in measurement. **b)** Radar charts of the focal pressures and discrepancies between measured and simulated values shows a lateral bias towards 45º and 90º that would not be captured in uncorrected simulations. Results are represented as mean ± SD.

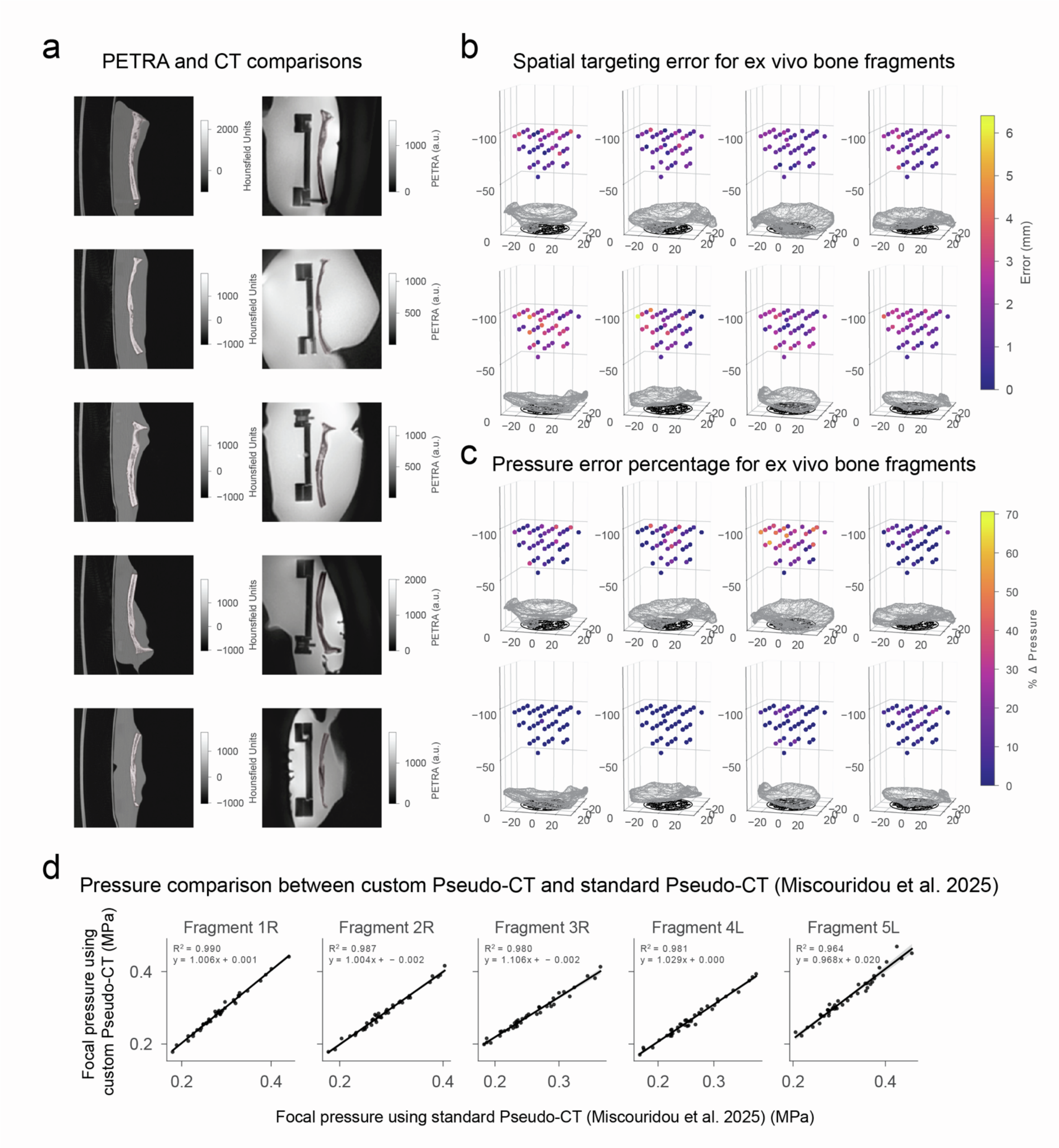


**Figure S4. Spatial targeting errors and simulated focal pressure errors through mounted temporal window skull fragments, Related to Figure 3.** **a**) Comparisons of CT and ZTE scans acquired for five fragments after rigid alignment between their bone masks. **b**) Depictions of each fragment with targets used for optimizing the bone alpha coefficient. Target points are color-coded by spatial error (mm) between intended and measured focal peak in the target plane. **c**) Similar depictions of the percentage error between measured and simulated target pressure at each point when using the optimized attenuation coefficient (15.3 dB/cm/MHz). **d**) For each of the five fragments, comparisons between the focal pressures from separately using the standard

Pseudo-CT fit (Miscouridou et al.[1]) or the custom Pseudo-CT fit confirms a highly linear relationship between the two mapping methods.

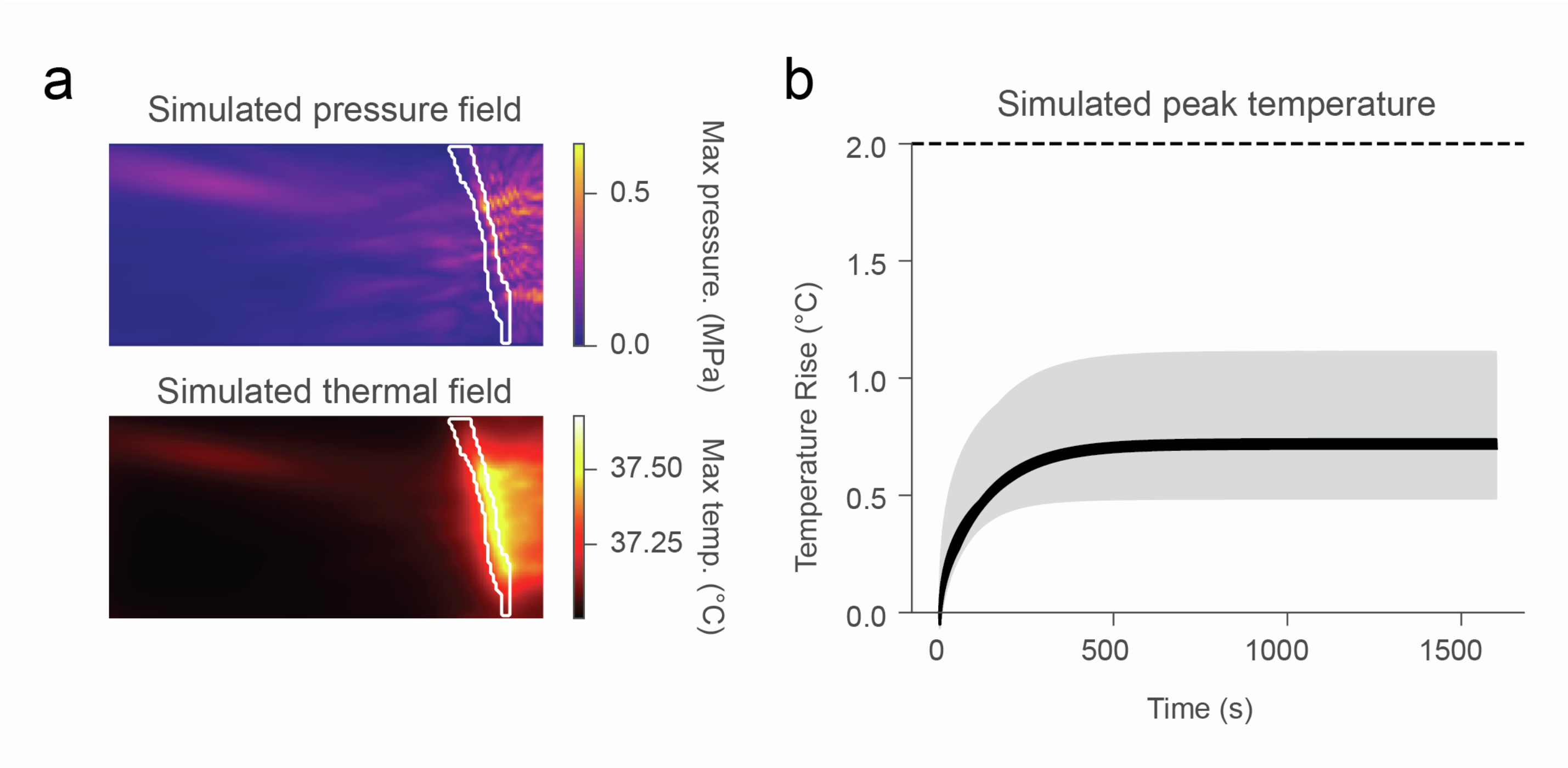


**Figure S5. Safety considerations when simulating worst-case temporal window temperature rise, Related to Figure 3.** **a**) Cross section of simulated pressure and thermal fields depicting max pressure and max temperature, respectively. White contours indicate the fragment bone mask. **b**) Simulated dynamics of maximum temperature rise in a bone fragment over the course of a 30-minute Pennes' bioheat simulation with bone attenuation coefficient of 15.3 dB/cm/MHz. Shaded band indicates range of peak temperatures when considering worst- and best-case attenuations in the range of 3.1 to 31.0 dB/cm/MHz.

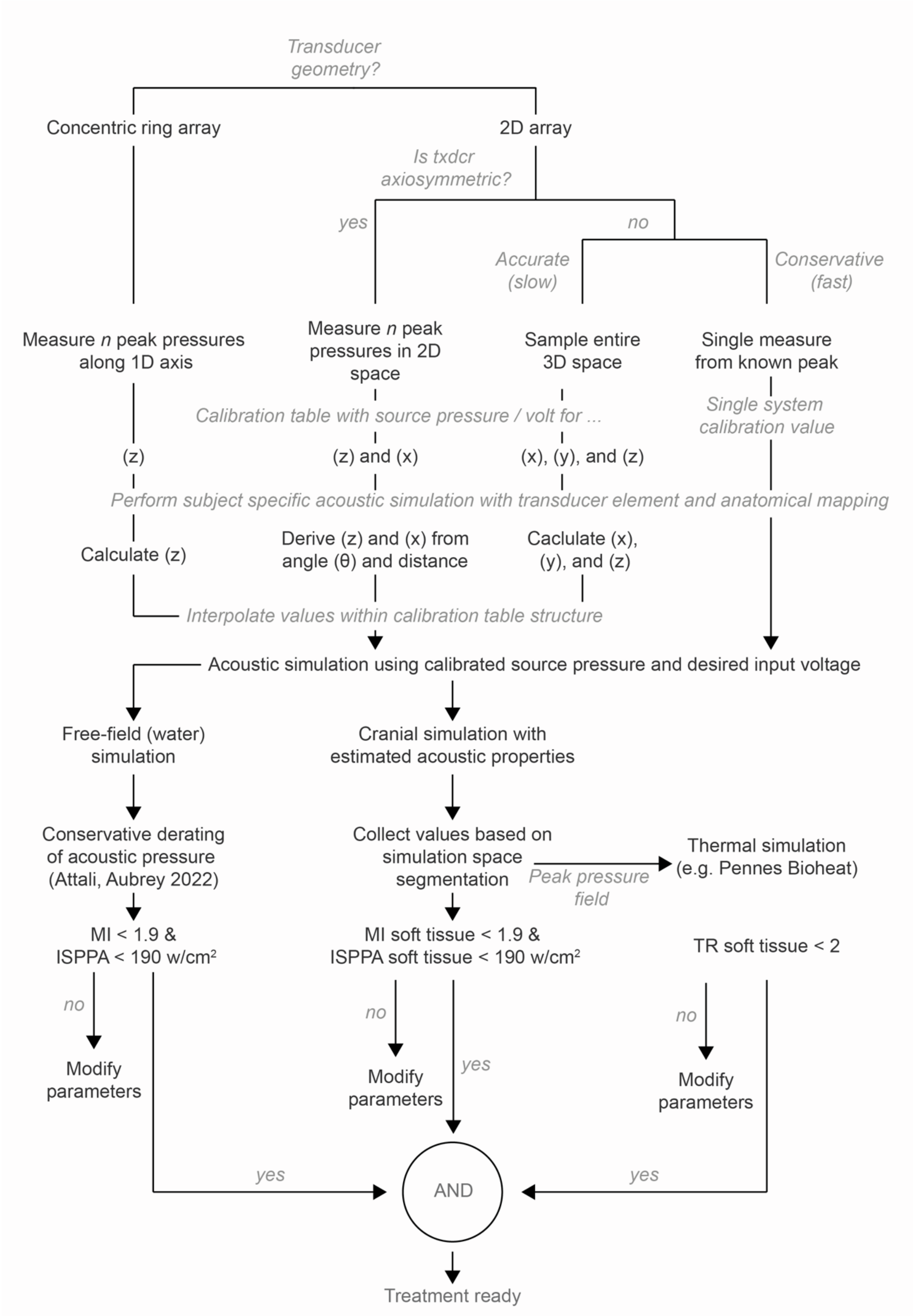


**Figure S6. Algorithmic approach to array calibration, Related to STAR Methods.**

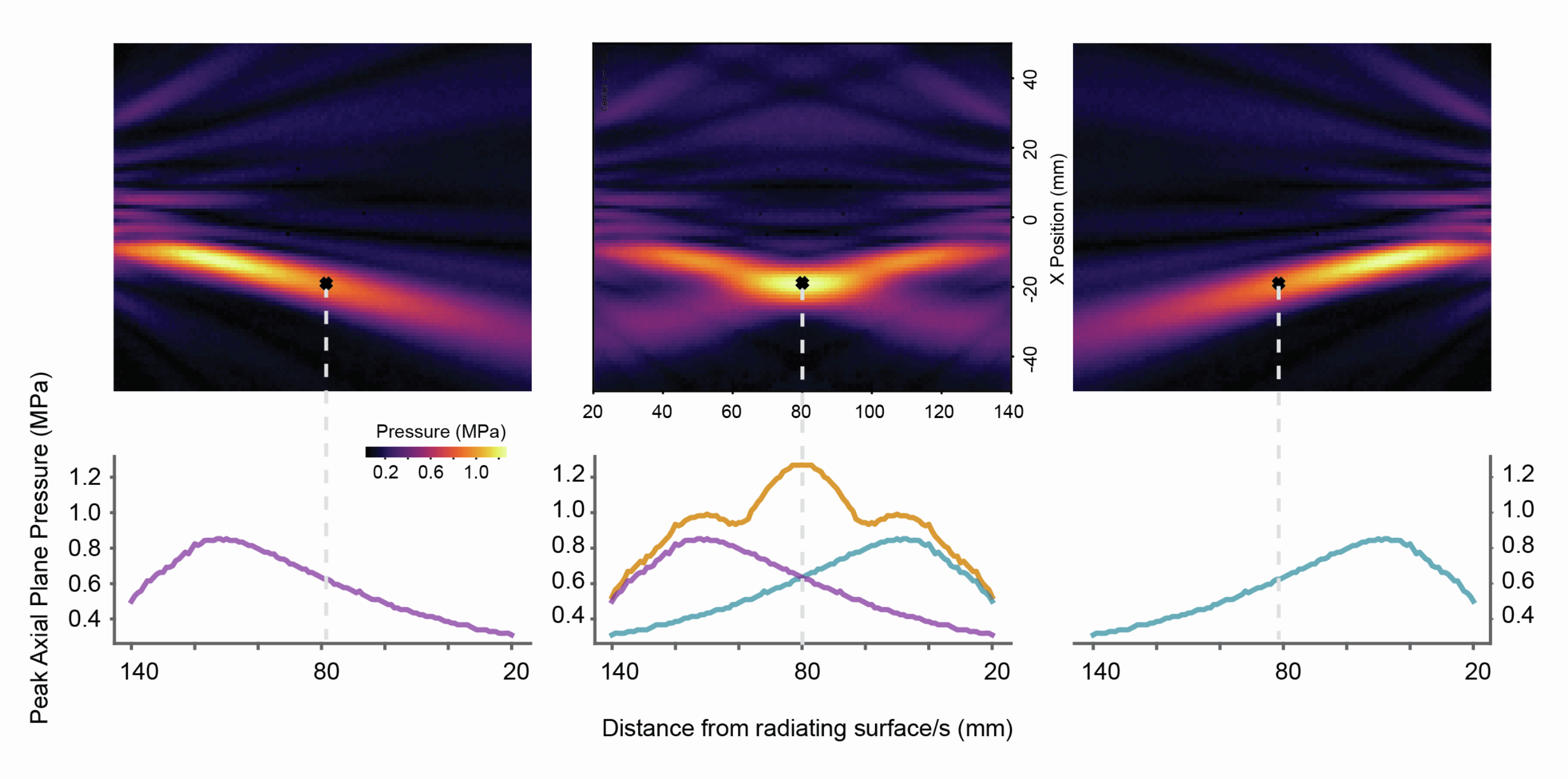


**Figure S7. Single array focusing may result in near-field shifted peak pressure, Related to STAR Methods.** Intersectional cross beams (Left, Right) where the intended focal positions are summed exceeds the peak pressure of independent near field pressures (Center).

## REFERENCES


1. Miscouridou, M., Krokhmal, A.A., Hess, A.T., Campbell, J., Stagg, C.J., Martin, E., and Treeby, B.E. (2025). Pointwise Encoding Time reduction with Radial Acquisition (PETRA) to pseudo-CT mapping for precision Focused Transcranial Ultrasound Applications. Preprint at arXiv, https://doi.org/10.48550/arXiv.2508.01050 https://doi.org/10.48550/arXiv.2508.01050.
