# Supplementary material for: Calibrated simulations for dynamic focusing of ultrasound through the temporal window
Source: Cell Rep Methods. 2026 Aug 14;6(10):101568. doi: 10.1016/j.crmeth.2026.101568 (PMC13721750; doi:10.1016/j.crmeth.2026.101568)
Supplement: Supplementary file 2 [file mmc2.pdf]

Cell Reports
Methods

Report

Calibrated simulations for dynamic focusing of ultrasound through the temporal window

Graphical abstract

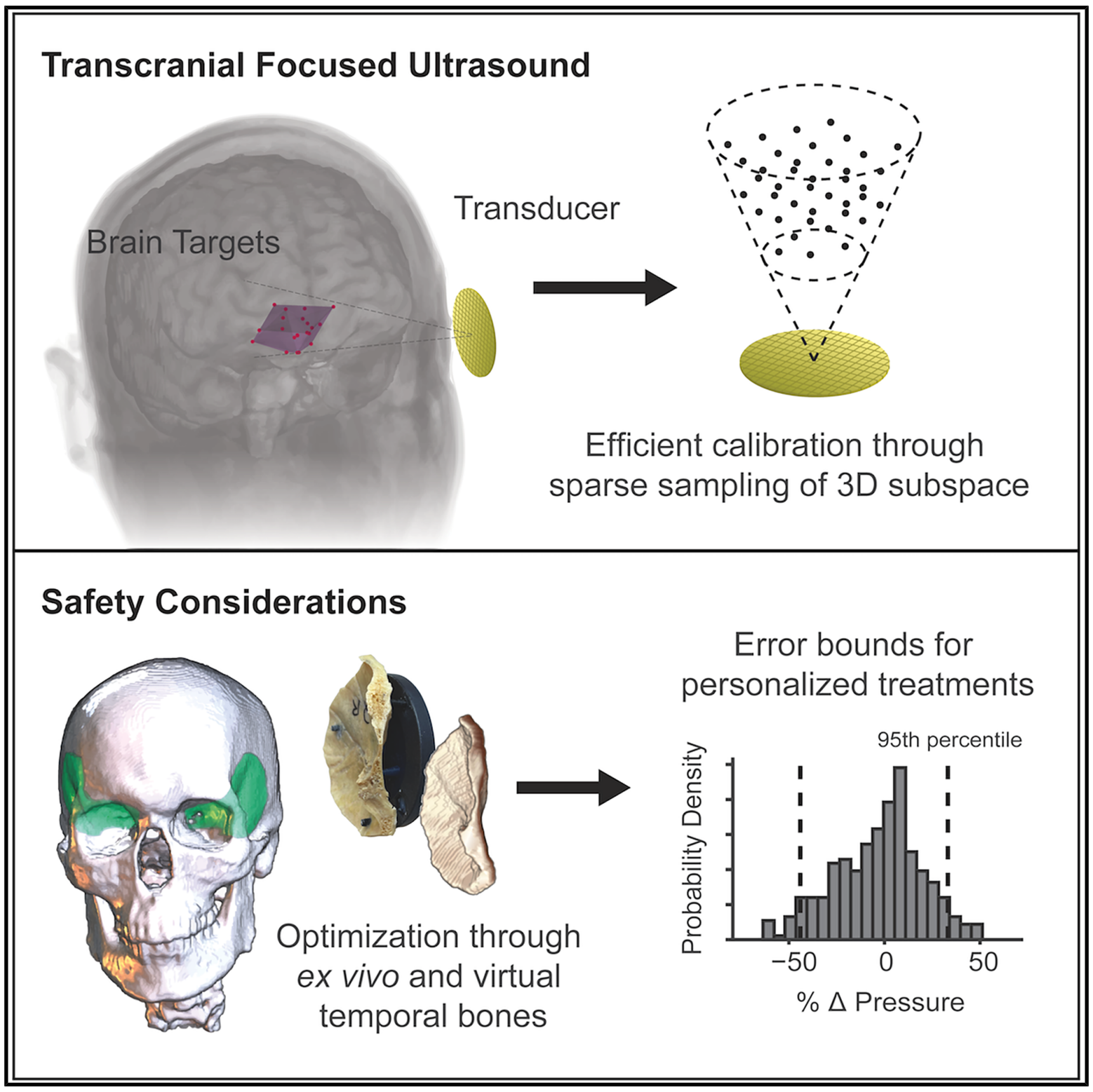

Highlights

- Sparse sampling can efficiently calibrate steering of transcranial ultrasound
- Calibration outperforms acoustic holography across a 3D steerable range
- Measurements through temporal window yield an optimized attenuation coefficient
- Error bounds enable worst-case assessment for personalized treatments

Authors

Ehsan Dadgar-Kiani, Vibha Hebbale, Malachy W. Newman, ..., Samuel Pichardo, Kim Butts Pauly, Keith R. Murphy

Correspondence

ehsan@attuneneuro.com (E.D.-K.),
keith@attuneneuro.com (K.R.M.)

In brief

Focused ultrasound can target deep brain regions but predicted and delivered pressures often diverge. Dadgar-Kiani et al. introduce a practical calibration framework based on sparse measurements and optimized skull attenuation that reduces this mismatch, enabling safer personalized transcranial ultrasound.

Dadgar-Kiani et al., 2026, Cell Reports Methods *6*, 101568
October 19, 2026 © 2026 The Authors. Published by Elsevier Inc.
https://doi.org/10.1016/j.crmeth.2026.101568

CellPress

Cell Reports Methods

CellPress OPEN ACCESS

Report

Calibrated simulations for dynamic focusing of ultrasound through the temporal window

Ehsan Dadgar-Kiani,[1,13,*] Vibha Hebbale,[1] Malachy W. Newman,[2] Grace Attalla,[1] John L. Alvarez,[1] Suraya Dunsford,[3,4] Kevin A. Caulfield,[5] Cameron H. Good,[1] Andrew D. Krystal,[6] Leo P. Sugrue,[6,7] Joline M. Fan,[6,8] Elsa Fouragnan,[3,4] Charles F. Caskey,[2,12] Samuel Pichardo,[9,10] Kim Butts Pauly,[11] and Keith R. Murphy[1,*]

[1]Attune Neurosciences, San Francisco, CA, USA
[2]Department of Biomedical Engineering, Vanderbilt University, Nashville, TN, USA
[3]Brain Research and Imaging Centre, Faculty of Health, University of Plymouth, Plymouth, UK
[4]School of Psychology, Faculty of Health, University of Plymouth, Plymouth, UK
[5]Department of Psychiatry, Medical University of South Carolina, Charleston, SC, USA
[6]Department of Psychiatry, University of California, San Francisco, San Francisco, CA, USA
[7]Department of Radiology and Biomedical Imaging, University of California, San Francisco, San Francisco, CA, USA
[8]Department of Neurology, University of California, San Francisco, San Francisco, CA, USA
[9]Radiology, Cumming School of Medicine, University of Calgary, Calgary, AB, Canada
[10]Hotchkiss Brain Institute, University of Calgary, Calgary, AB, Canada
[11]Department of Radiology, Stanford University, Stanford, CA, USA
[12]Department of Radiology & Radiological Sciences, Vanderbilt University Medical Center, Nashville, TN, USA
[13]Lead contact
*Correspondence: ehsan@attuneneuro.com (E.D.-K.), keith@attuneneuro.com (K.R.M.)
https://doi.org/10.1016/j.crmeth.2026.101568

MOTIVATION Acoustic simulations are used to plan and assess the safety of transcranial focused ultrasound for neuromodulation, but predicted focal pressures often significantly diverge from measured values and can be heavily dependent on the focal location. Existing calibration approaches are both impractical at scale and location blind. We aimed to develop a practical framework for resolving simulation-measurement mismatch that provides explicit error bounds to support safer personalized neuromodulation.

SUMMARY

Focused ultrasound can be delivered through the temporal window to modulate heterogeneously located brain areas. Acoustic simulations allow for safety assessments when dynamically targeting brain structures, but the mismatch between simulation and measured focal pressure can vary across the steerable range due to mechanically inaccurate assumptions made about the skull and transducer. Here, we describe efficient methods for simulation-measurement calibration using axisymmetric projections and sparse sampling across a 3D steerable subspace encompassing deep brain targets across 157 subjects. To address the simulation-reality mismatch in skull transmission, we used the measured and predicted pressure values through eight human temporal window fragments to derive an optimized bone attenuation coefficient. Collectively, the calibration framework and optimized temporal window coefficients can be used broadly across studies to improve the accuracy of reporting and dependent safety assessment for personalized neuromodulation treatments.

INTRODUCTION

Ultrasound neuromodulation is a rapidly emerging brain stimulation technology being actively researched and developed for therapeutic clinical applications[1–6] and causal functional brain investigation.[7–11] As ultrasonic modulatory technology develops, a key consideration is how simulations of pressure and temperature rise can inform the safety and accuracy of beam steering capabilities of multi-element transducers.[12–14] Researchers often use transducers with a pre-defined and often fixed focal position, allowing for a single calibration value between the transducer drive voltage and focal pressure in free-field conditions.[12]

In contrast, multi-element arrays in a two-dimensional (2D) plane allow for dynamic steering of an ultrasound focus across three-dimensional (3D) space. With large mechanical or electrical crosstalk, there may be substantial errors in simulation unless differential calibration is used when steering to different locations

This is an open access article under the CC BY license (http://creativecommons.org/licenses/by/4.0/).

in 3D space.[15–18] More broadly, these simulation-measurement mismatches reflect two distinct categories of uncertainty: device and simulation-related factors, such as element crosstalk or element directivity, and patient-specific factors, such as skull microstructure and heterogeneity. Accurate characterization of the first category is a prerequisite for patient-specific modeling, because otherwise device-level discrepancies would be confounded with subject-level effects. If the location of the array is known relative to the subject's anatomy, an approach of calibration for every point in space can be used to target heterogeneous brain space in a highly precise manner (Figure 1A).

While this capability extends researchers' placement and targeting capabilities, highly sampled voltage-pressure maps across a given steering range may be impractical, particularly if each independent ultrasound system requires its own calibration. Although it is possible to characterize fields based on exact relative coordinates derived from individual modeling, logistical challenges arise when the device is deployed in the field and target position varies across each use. To address these challenges as the use of focused ultrasound (FUS) neuromodulation scales, we examined the relationship between spatial calibration resolution and accuracy within a single plane and in 3D space.

RESULTS

We first determined the spatial extent of FUS targeting by extracting the relative target coordinates from four bilateral deep brain targets: the caudate, amygdala, centromedian nucleus of the thalamus, and subthalamic nucleus across 157 subjects. Since the 2D array coverage has a circular aggregate symmetry, the coordinates were then flattened into a single plane, capturing the axial and lateral steering depth by zeroing the azimuthal angle (Figure 1B). The calibration space was then defined as the truncated cone that captured 95% of the target coordinates in the axial and lateral domains. In principle, this statistical approach to defining calibration space can be applied to any study where a set of brain target coordinates is known relative to a transducer, which may narrow or broaden calibration space. Initially, we examined whether acoustic holography could be used to create source pressure patterns sufficient for creating accurate simulation of pressure outputs with our array. As described previously,[18] the phase and amplitude for each array element was independently collected at a depth of 30 mm from the transducer radiating surface. The plane was then backpropagated to derive a source pressure mapping for each element (Figure S1A). Using the reconstructed pressure sources for each element yielded significant mismatch between measurement and simulation peak pressures across the steerable range in excess of 20% (Figure 1E). This mismatch is likely due to large crosstalk of elements that is not accounted for when measuring element activity in isolation.[17,19] Thus, while acoustic holography may be sufficient for dynamically steered focal calibration in arrays with highly isolated elements, partially isolated bulk piezoelectric arrays may require direct measurement across the steering space.

To directly build a mapping between simulation and measurement, we captured the peak focal pressure in simulation and measured field when steering to fixed locations across the steering range. The peak was chosen rather than intended focal position even in the event that the two were slightly offset. This ensures that the calibration is conservatively safe by assuming the plane's maximum pressure. To identify a minimal and practical sampling frequency needed to characterize a 2D calibration space, the steering range was initially sampled at the Nyquist spatial frequency of 1.5 mm based on the free-field wavelength of ~3 mm at 500 kHz. For all positions, the hydrophone scan was set to sample the focal plane at 6 points per wavelength, below Nyquist frequency. The simulated focal pressure was then compared with the experimentally measured focal pressure, determined by taking the peak within the focal target's axial plane (Figure 1C). The focal pressure obtained when steering the array to all coordinates in the calibration space defines a focal pressure mapping, and can be constructed from both simulated and experimental focal pressures (Figure 1D). The difference between these two maps defines the discrepancy between simulated and measured target pressures, and contained a gradual, low-frequency component with the near-field lateral target points showing the largest discrepancy. The data were well captured with an anisotropic Gaussian function fit, and recomputing the focal pressure discrepancies after applying the correction function was shown to be drastically more effective in reducing pressure differences than holographic correction (Figure 1E). Using random stepwise subsampling of target pressure discrepancies across the calibration space, we found that 50 points produced a strong fit (R^2 = 0.83) with negligible improvements from increased sampling (Figure 1F). Fifty points correspond to an equidistant spacing of 6 mm between sampled points within the calibration space. Going forward, we utilized an equidistant 6-mm spacing of target points when calibrating 2D planes and 3D cones. Also, given that we observed similar distributions of spatial frequencies in simulated focal pressure maps across concentric circles, Fermat spirals, and normally distributed element grid designs (Figures S1A and S1B), we expect that this simulation-reality correction for focal pressure maps can be applied to a multitude of array designs.

In theory, arrays with axial symmetry may also contain symmetric crosstalk and calibration fields, and different coordinates with conserved depth-angle along that axis should produce highly similar pressure profiles. In this case, a single plane could theoretically be measured as a proxy for planes rotated along the orthogonal axis. As expected, acoustic simulations produced largely axisymmetric pressure fields for both axisymmetric and asymmetric arrays. However, modest differences were found when experimentally measuring 8 planes' focal pressure maps at 45° azimuthal steps for our random non-axisymmetric array, potentially due to manufacturing variability or crosstalk not captured by the simulations (Figures 1G and S3). In the event that these planes are largely conserved, or that the relationship between them is well characterized, a 2D plane may be rotated around the axis of symmetry to produce a calibration value for points in 3D space. However, in the event of plane mismatch, a 3D characterization is necessary for each transducer. To identify a minimum viable number of points, we sampled the entire 3D steerable range at 6-mm resolution (Figure 1H). Based on automatic elbow detection of R^2 fit, we found that the 3D space was well fit with just 20 points (Figure 1I), likely due to the low spatial frequencies composing the discrepancy between simulation and

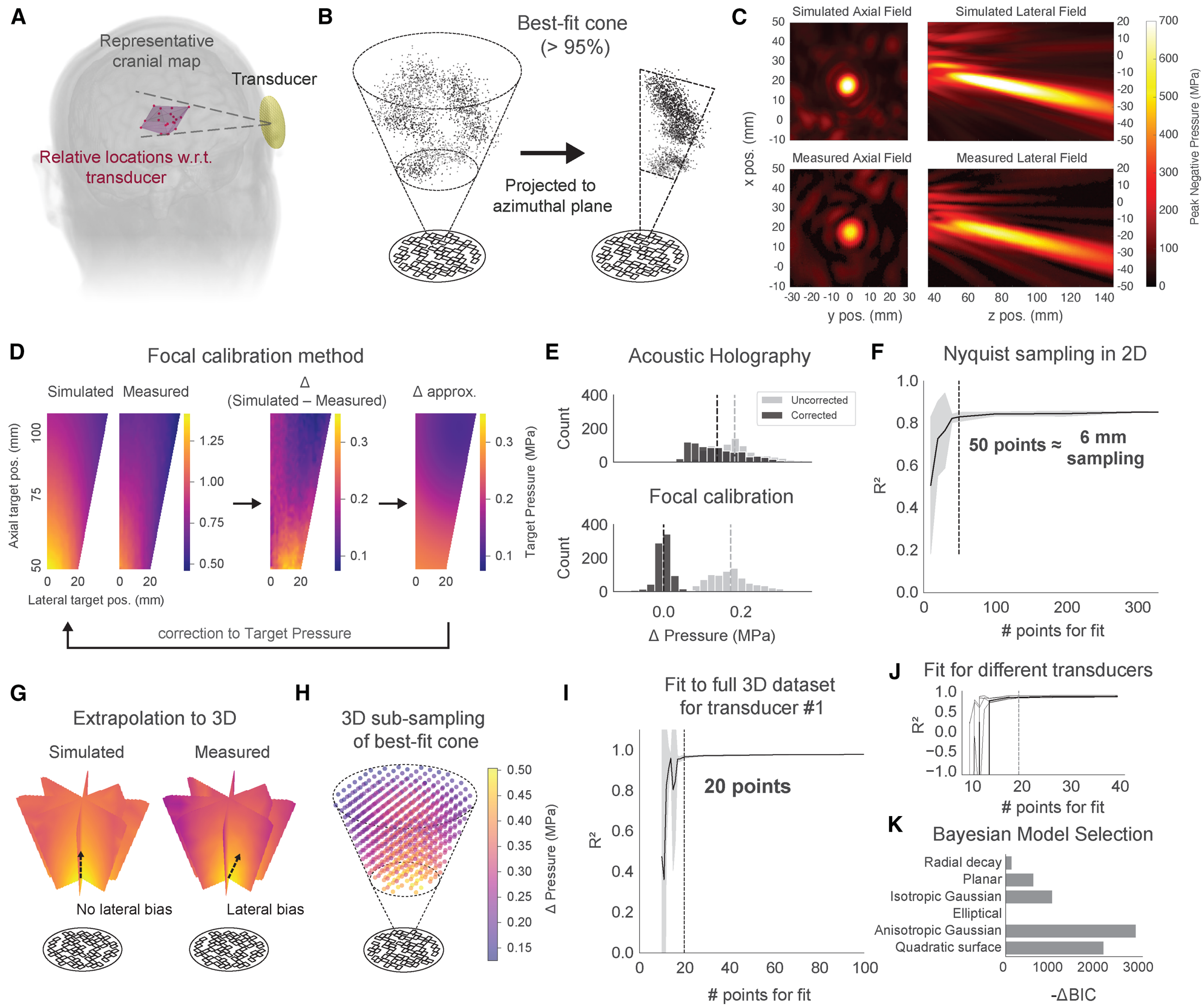

Figure 1. 3D steering simulation calibration for FUS neuromodulation

(A) Target coordinates relative to a single transducer array across heterogeneous individual anatomy.

(B) Target coordinates in 3D space (left) can be flattened (right) to represent axial and lateral distances within an azimuthal calibration plane.

(C) Comparisons between axial and lateral planes of simulated and measured ultrasound pressure fields.

(D) A schematic of the focal correction method. Difference between simulated and measured focal pressure maps at Nyquist-sampling across the calibration plane shows a low-frequency gradient mismatch, which can be approximated by a global fit and used to correct a simulation's focal pressure while steering within the calibration plane.

(E) Compared with acoustic holography, the focal calibration method better minimizes the differences between simulated and measured focal pressure.

(F) Within the Nyquist-sampled calibration plane, anisotropic Gaussian function fit strength related to equidistant number of points sampled for fitting the mismatch (Δ) between 2D focal pressure maps from simulation and measurement. Results are represented as mean ± SD.

(G) Planar measurements of focal pressure at different azimuthal angles show non-uniformity not evident in simulated planes. Arrow indicates the lateral spatial bias observed in measurements.

(H and I) Visualization of 3D calibration field fully sampled at 6 mm (H) is well fit by a 3D anisotropic Gaussian function (I). Results are represented as mean ± SD.

(J) Similar fits with comparable convergence patterns around 20 points were obtained for other transducers ($N = 5$).

(K) Bayesian model selection can be used to identify the appropriate model type that captures the simulation-reality mismatch without unnecessary complexity.

See also Figures S1, S2, and S3.

measurement. This approach also generalized to five other transducers with the same element configurations (Figures 1J and S2A), with each showing similar convergence patterns of fit at just about 20 points (Figures S2B and S2C). Using Bayesian model selection, other functions used to fit the data underperformed relative to the 3D anisotropic Gaussian function (Figure 1K). While automatic elbow detection balances accuracy with efficiency and diminishing returns from adding more points, other objective methods for selecting a sampling rate may also be used, such as a mean discrepancy threshold.

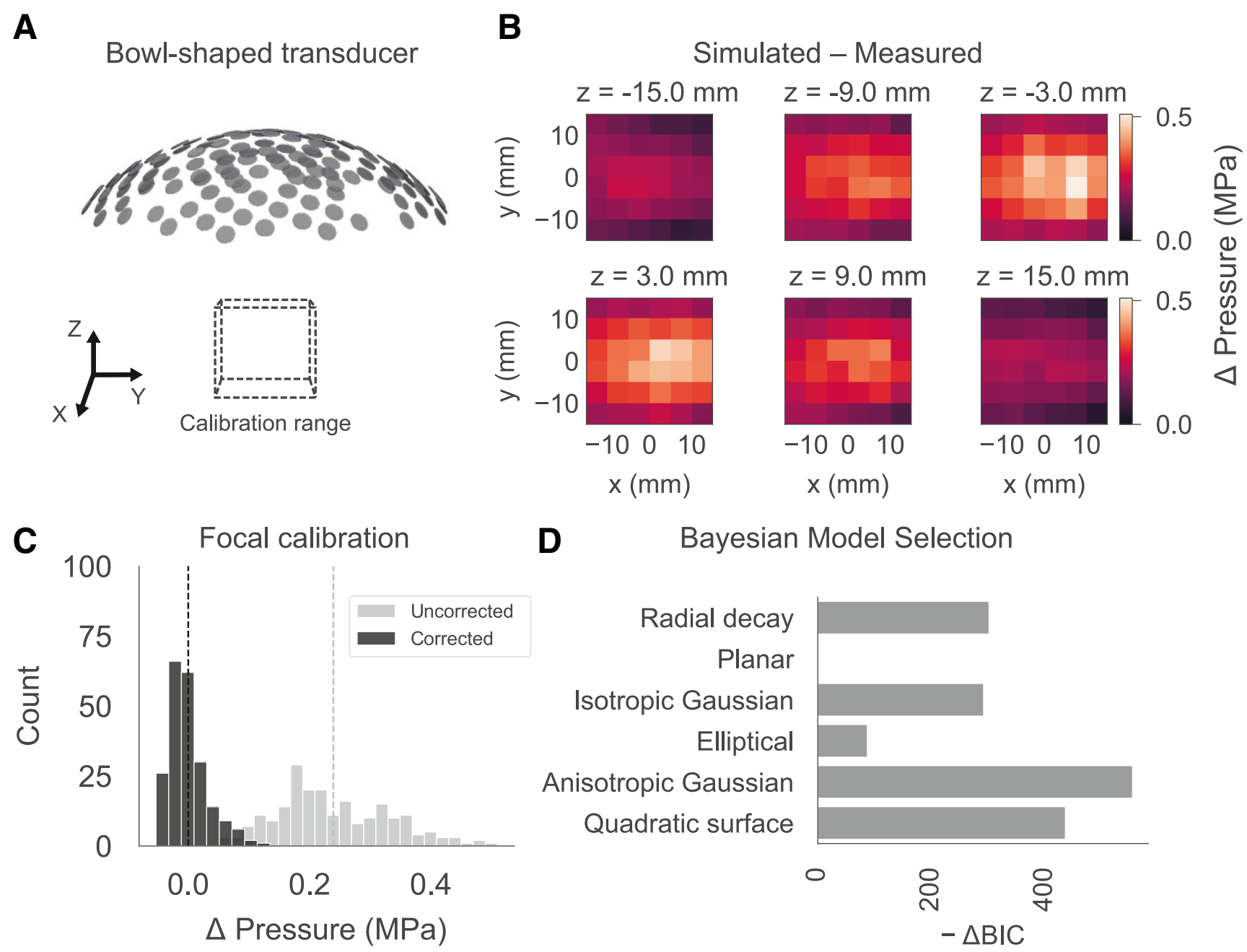

Figure 2. Evaluation of 3D focal calibration method for bowl-shaped transducer geometry

(A) Depiction of the bowl-shaped transducer used for both hydrophone and simulated focal pressure measurements at each grid point within a calibration range centered at the geometric focus of the bowl.

(B) Discrepancies between simulated and measured focal pressure maps depict largest differences near the geometric focus with apparent radial decay.

(C and D) Application of the focal calibration method with an anisotropic Gaussian function can minimize differences between simulated and measured focal pressures across the entire calibration range (C), with similar functions (anisotropic Gaussian and quadratic surface) demonstrating generalizability through Bayesian model selection across both the random element planar transducer and the bowl-shaped transducer (D).

To validate that this calibration method may be used with other array designs and drive systems operating at different frequencies, it was also tested on a bowl-shaped transducer with a Fermat spiral geometry driven at 900 kHz (Figure 2A). The calibration range was centered at the geometric center of the transducer and spanned 30 mm in all dimensions. Target coordinates were selected by sampling this range at 5 mm resolution and comparing the measured focal pressure when targeting each of these coordinates with the simulated focal pressure yielded a 3D discrepancy map (Figure 2B). The largest discrepancies were located at the geometric center and decayed radially outward. The focal calibration method was applied to these discrepancies using an anisotropic Gaussian function and captured the data well (Figure 2C), much like with the custom sparse 2D array driven at 500 kHz. Bayesian model selection yielded similar top-ranking model as with the 2D array (Figure 2D), such as an anisotropic Gaussian and quadratic surface.

The calibration method described can be applied in simulations to approximate the output of ultrasound neuromodulation devices in water, which closely approximate the acoustic properties of the brain. As both water and brain tissue are highly transmissive of ultrasound, pressure measurements in water can generally be assumed to represent the maximal pressure in the brain, assuming that the skull is acoustically transparent and the absence of standing waves from the skull interior.[18,19] In contrast, the change in acoustic properties as waves interact with the skull can induce appreciable phase shifting, absorption, reflection, and scattering, substantially altering ultrasound transmission. Lower resolution computed tomography (CT) or magnetic resonance imaging (MRI) of gross bone structure can allow for coarse estimation of these features. However, cortical bone contains finer structural elements that are not practically observable with imaging.[20] In particular, the trabecular bone has numerous heterogeneous cavities with rapid changes in acoustic impedance, which can result in substantial scattering. Indeed, earlier works demonstrated that the majority of ultrasound attenuative losses across calvarial bone are from scattering rather than absorption.[20] Given the practical resolution limits of current imaging and simulation frameworks, microstructure scattering cannot be properly simulated and attenuation by scattering is heavily underestimated. A workaround to this issue is the addition of a loss term to acoustic simulation space assigned specifically to bone. Within pseudospectral time-domain simulations, as implemented formally by k-wave,[21] attenuation coefficients are modeled with a frequency power-law $\alpha(f) = \alpha_0 f^y$ together with the matched dispersion term implemented via fractional-Laplacian operators. Using a viscoelastic full-wave model as implemented formally by BabelBrain,[22] attenuation and dispersion are modeled by viscoelastic constitutive laws with relaxation or Q-factor terms, assigning longitudinal and shear losses per voxel from CT-derived property maps. While these terms are effective at representing loss, accurate assignment accounting for bone loss has not yet been determined.

To optimize and understand the attenuation associated with dynamic steering through the temporal window, we first approximated the device position on each of five cadaver skulls. We temporarily placed 5 mm silicone pads between the device and bone to mimic the soft tissues of the scalp which are absent on the skull. Using a guide mock transducer, we measured the distance from mock transducer fiducials to the temporal window bone and marked the relative orthogonal projection point. The mock transducer was then fitted with posts to replicate the depth to skull while a surrounding bracket was mounted to the skull (Figure 3A). Both left and right temporal windows were then cut from the skull, degassed in water, and scanned with a zero echo time (ZTE) MRI sequence as previously described[23]; this process was performed for each of 5 skulls, yielding 8 different skull fragments after 2 fragments were discarded due to

CellPress
OPEN ACCESS

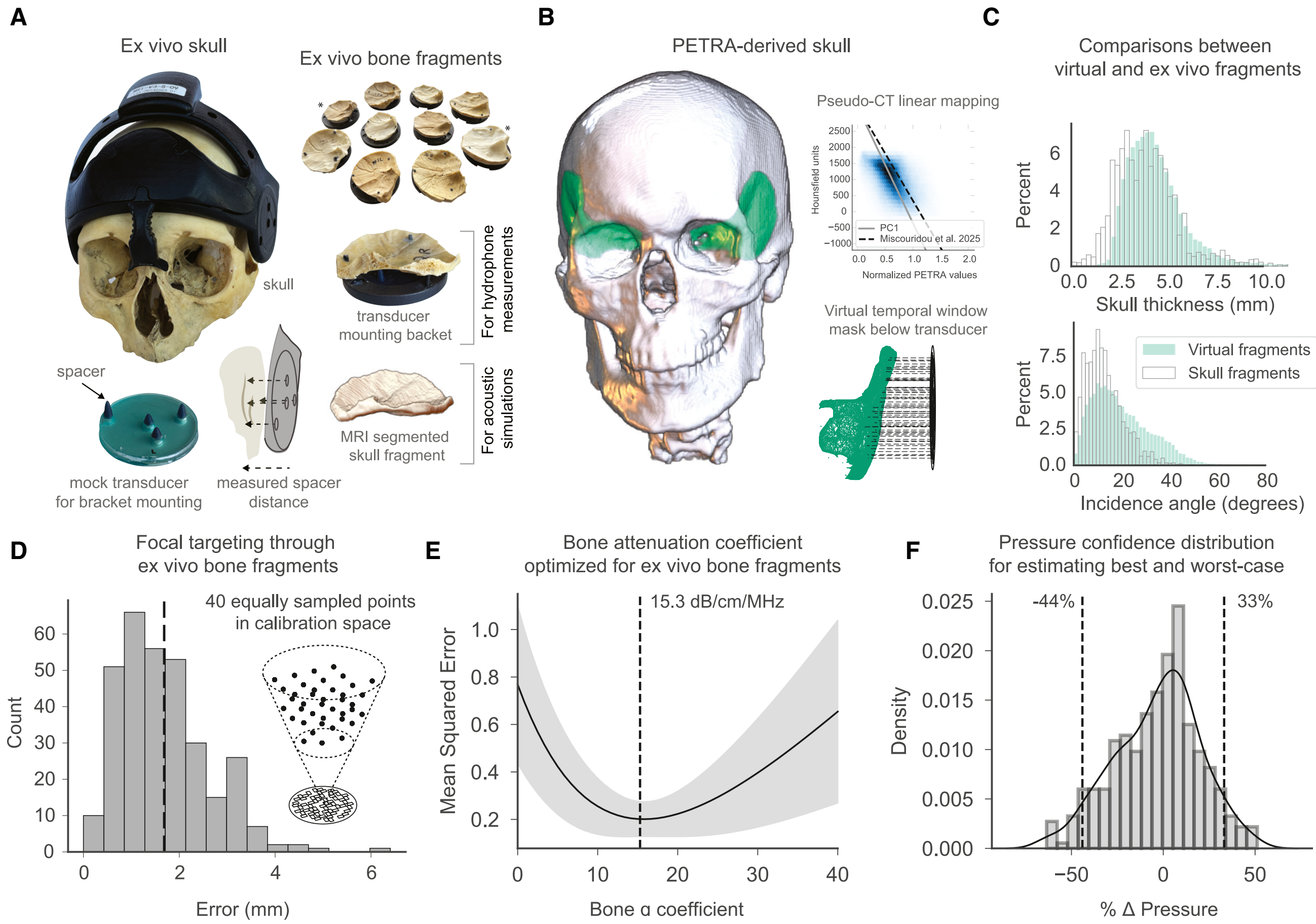

Figure 3. Optimization of simulation attenuation with heterogeneous temporal window transmit measurements

(A) Wearable device is placed on each human skull sample for measurement of distance from transducer control points to temporal window. A mock transducer with those recorded distances set with spacers is used to further mount a bracket for holding the ultrasound transducer in a fixed location relative to the skull. The bracket and transducer location are captured in the MRI space of each skull fragment. Image of mounted temporal window skull fragments. Asterisks indicate two fragments that were omitted due to damage.

(B) Normal projections from each transducer element to ZTE-derived skull masks were used to extract "virtual" temporal windows. An example virtual temporal window is shown. CT and ZTE scans of five bone fragments were also used to derive a linear relationship between Hounsfield units and ZTE voxel units, in close agreement with mappings in previous literature.

(C) Population distribution of virtual temporal windows extracted from 157 subjects is compared with simulated projections from *ex vivo* skulls for element angle of incidence and temporal window thickness.

(D) Focal targeting was performed at 40 equally sampled points in the calibration space for each *ex vivo* bone fragment, and the histogram depicts the spatial targeting error (mm).

(E) Plot of mean squared error between measurement and simulation focal pressure with stepwise homogenous assignment of ultrasound wave attenuation in bone. Error intervals indicate standard deviation in mean squared error across all fragments and focal targets for a given attenuation coefficient.

(F) At the optimal attenuation coefficient (α = 15.3 dB/cm/MHz), the 95th percentile overestimation of focal pressure corresponds to a 33% increase in pressure, while the 5th percentile underestimation corresponds to 44% decrease. See also Figure S4.

cracking damage along the sutures (Figure 3A). To examine whether our fragments were representative of live subjects' scanned temporal windows, we used each subject's ZTE scan and the transducer elements' orthogonal projections to virtually extract both the left and right temporal windows from 157 subjects' MRI scans (Figure 3B). The *ex vivo* fragments closely resembled the virtual temporal windows in both skull thickness and incidence angle as measured through each transducer element's projection (Figure 3C). This indicates that findings and simulations performed with the bone fragments would be applicable to live subjects.

For each fragment, element phase delays were calculated through acoustic simulation time reversal targeting each of 40 locations equally sampled across the calibration space (Figures 3D, S4B, and S4C). The mean targeting error across each combination of 8 bone fragments and 40 spatial targets was found to be 1.68 ± 0.96 mm off from the intended target (Figure 3D). Irrespective of the targeting error, we extracted the peak pressure from the target plane to ensure conservative safety calibrations. We then compared these measured pressures with simulated peak pressures obtained from forward acoustic simulations as we incrementally modified the

homogenous bone attenuation coefficient (α_0). The mean square error between simulation and reality was minimized with a value of 15.3 dB/cm/MHz (Figure 3E), in close alignment with previous experimental estimations of skull bone attenuation of 13.3 dB/cm and 16.6 dB/cm at 1 MHz.[20,22] To derive conservative boundaries when using this optimized value, we calculated the 5th and 95th percentile pressure error across simulation-measurements. The 5th percentile overestimation and 95th percentile underestimation found that the pressure could be 44% lower or 33% higher, respectively, than the simulated value (Figure 3F).

Although this study emphasizes estimation of pressure and mechanical index, thermal derivatives are also important for ultrasound neuromodulation safety.[14] The previously mentioned under and overestimations in pressure have important implications in estimating the range in thermal rise, as thermal rise is driven proportionally by intensity, which is in turn proportional to the square of the pressure. Given that there are many additional parameters that may guide thermal dynamics, such as pulse duty cycle and total, we provide only a representative example of how to estimate the bounds of temperature rise bone under a certain set of parameters (Figure S5A). In this example operating at 5 Hz with 10% duty cycle for 30 min, thermal simulations were performed where the calibrated pressure field was derived and passed into the Pennes' bioheat equation. Based on previous works, the absorption was set to 16.8% of attenuative losses[20] with any remaining fraction of attenuation beyond 16.8% likely represents scattering. Importantly, an attenuation coefficient was tested to the corresponding to the 5th percentile underestimation of transmission to the target coordinate (31.0 dB/cm/MHz), as this would in turn overestimate the absorption within the bone. The range of maximum temperature rises across attenuation values ranging from 3.1 dB/cm/MHz (95th percentile overestimation of transmission) and 31.0 dB/cm/MHz (5th percentile underestimation of transmission) were calculated and compared with the relevant safety threshold for temperature rise in bone (+2°C), as depicted in Figure S5B.

DISCUSSION

In this study, we provide a methodology for calibrating simulation of ultrasound arrays foci across a steering range. We further optimize simulation attenuation to improve simulation accuracy and to understand simulation error boundaries with respect to peak pressures. This will ultimately improve ultrasound neuromodulation reporting and our understanding of safe stimulation parameters. Devices that lack steering capability or have a preset number of focal positions may have simpler calibration requirements, such as making a single measurement per device. Furthermore, a device with measured axial symmetry may require less measurement. While non-definitive, we provide a suggestive algorithm for calibration steps depending on the transducer type (i.e., single vs. multi-element) (Figure S6). For skull attenuation, we optimize for accuracy and include a distribution of estimates to reflect the heterogeneity in bone microstructure that impacts ultrasound transmission. This approach improves upon our earlier practices, which reported single, conservative estimates that consider the skull as highly transmissive,[2] which may be useful in the absence of skull information.[24] In this way, variant methods can be purposed toward capturing more realistic values rather than conservatively high values. Nevertheless, methods of attenuation use should be clearly specified in the methodology to properly guide replication efforts and safety.[13] Importantly, because the optimized skull attenuation coefficient operates as a lumped parameter and does not resolve losses at the simulation grid resolution, its value is specific to the simulation framework used to derive it. Groups using finer spatial grids, different absorption power-law exponents, or viscoelastic solvers with shear-wave handling should anticipate different optimal values and may need to rederive it against their own measurements.

In addition to the data-driven approach to calibration field size creation used here, other viable methods may include population-level biometric data based on head anatomy,[25] or large MRI datasets.[26–28] For instance, bizygomatic breadth, or the maximum width measured between the outer edges of the cheekbones, has been captured for large sample populations using calipers which offer 5th percentile and 95th percentile ranges for both sexes.[25] Furthermore, field size may be determined by an acceptable ratio between the main lobe and side or grating lobes present.[12]

That the focal correction method with an anisotropic Gaussian strongly recovers the simulation-measurement mismatch for two transducers with very different geometries, element counts, and operating frequencies (a custom 64-element sparse 2D array at 500 kHz and a 128-element Fermat-spiral bowl array at 900 kHz) is consistent with the observation that the discrepancy is dominated by low spatial frequencies and smoothly varying error sources. These errors may include element directivity falloff away from the geometric focus and cumulative mechanical or electrical crosstalk that are not included in standard simulation methods. Thus, this correction method is likely to generalize broadly to other multi-element arrays after just a brief sampling of their respective steering spaces.

Regardless of the methodology used for calibration, discrepancies between the intended and measured focal position may arise from subtle mechanical registration error, or element phase distortion associated with device crosstalk (Figure 1C). Thus, capturing the actual peak rather than taking a direct measurement at the intended focus is strongly recommended to ensure that the calibration conservatively accounts for the largest possible biomechanical effects. This approach enables the accurate assessment of safety limits derived from peak pressures such as Mechanical Index (MI) or spatial peak pulse average intensity (I_{SPPA}). In some cases, the focus may contain secondary fields likely associated with transducer element mechanical crosstalk stemming from limited physical isolation. This crosstalk may shift the peak pressure closer to the array face than intended, despite conservation of steering angle. If there is only one stimulation focus, then the axial plane may be used to determine the appropriate lateral plane for capturing the calibration focal peak. In the event that multiple foci are targeted to a cross-beam location arising from multiple transducers, the peak plane will be at the intended axial target plane (Figure S7) at the superposition of the two beams, as implemented by numerous teams.[2,29,30] In these instances, measurement of the peak at the intended focal plane may be advisable to avoid reporting overestimated peak pressures. Regardless of configuration, the measured field volume

should be sufficiently large to capture the focal peak, where the size should be expanded until the field maximum value is not found at the edge of the measurement window. Furthermore, the expected pressure from the calibration must be accounted for within the source pressure assignment, or within a multi-stage error assessment that also includes biological inputs to the system, such as the skull.

Although this work emphasizes estimation of pressure and mechanical index, thermal derivatives are also important for ultrasound neuromodulation safety.[11] The example depicted in Figure S5 offers one possible method for considering worst-case temperature rise in skull bone due to pressure changes, although considerably more work is required to explore the dependence on variable absorption and specific ultrasound driving conditions. The calibration framework presented here is intended as a prerequisite, rather than a replacement, for patient-specific modeling, as it will establish a device-level baseline upon which patient-specific simulations of skull transmission and downstream safety estimates can be reliably built. Collectively, these methodologies will improve the accuracy of simulations and thus, the assumptions of safety when using steerable ultrasound arrays for human applications.

Limitations of the study

A limitation of note in this study is that our proposed focal calibration method only aligns the mismatch between the simulated and measured peak focal pressures. Any other discrepancy, both within the focus and in the surrounding field, for instance, due to side or grating lobes, is not addressed. Although this limitation is unlikely to affect most safety considerations where peak pressure is mostly considered, there may be applications where specific off-target pressures would need to be minimized, and their corresponding simulation-reality mismatches need to be better characterized. Nonetheless, the focal calibration method introduced here is complementary to other methods that would aim to correct beyond the focal pressure, which future studies may aim to do.

There are also several important limitations regarding our optimization of the attenuation coefficient for steering through bone. Firstly, the eight *ex vivo* temporal window fragments used for hydrophone measurements may not represent the variability in skull microstructure. Considering that skull microstructure may vary with demographics and that the donor demographics were not reported, it is difficult to assert the universality of this optimized parameter. In addition, due to the lack of *in vivo* validation, further attenuating effects arising from the scalp, perfusion, or intracranial fluid may affect the 5th–95th percentile pressure error band. However, the conservative worst-case attenuation presented here remains a reasonable upper bound for safety assessments, and future studies using direct measurements of intracranial pressure may seek to tighten this error bound.

RESOURCE AVAILABILITY

Lead contact

Requests for further information and resources should be directed to and will be fulfilled by the lead contact, Ehsan Dadgar-Kiani (ehsan@attuneneuro.com).

Materials availability

This study did not generate new unique reagents.

Data and code availability

- Raw data are available from the lead contact upon request.
- All original code has been deposited in GitHub (https://github.com/dadgarki/fus_safety_paper) and Zenodo (https://doi.org/10.5281/zenodo.20840906) and is publicly available as of the date of publication.
- Any additional information required to reanalyze the data reported in this paper is available from the lead contact upon request.

ACKNOWLEDGMENTS

This work was supported by Attune Neurosciences, Inc.

AUTHOR CONTRIBUTIONS

Conceptualization, E.D.-K. and K.R.M.; methodology, E.D.-K., C.F.C., S.P., K.B.P., and K.R.M.; investigation, E.D.-K., V.H., M.W.N., G.A., and J.L.A.; software, E.D.; writing – original draft, E.D.-K. and K.R.M.; writing – review and editing, E.D.-K., M.W.N., G.A., S.D., K.A.C., C.H.G., A.D.K., L.P.S., J.M.F., E.F., C.F.C., S.P., K.B.P., and K.R.M.; resources, E.D.-K., V.H., M.W.N., G.A., J.L.A., L.P.S., C.F.C., and K.R.M.; funding acquisition, C.F.C. and K.R.M.; supervision, E.D.-K. and K.R.M.

DECLARATION OF INTERESTS

E.D.-K. is an employee of and holds stock in Attune Neurosciences, Inc. V.H. is an employee of and holds stock in Attune Neurosciences, Inc. G.A. is an employee of Attune Neurosciences, Inc. J.L.A. is an employee of and holds stock in Attune Neurosciences, Inc. C.H.G. is an employee of and holds stock in Attune Neurosciences, Inc. E.F. serves as a scientific advisor to Attune Neurosciences, Inc. K.B.P. serves as a scientific advisor to Attune Neurosciences, Inc. K.R.M. is an employee of and holds stock in Attune Neurosciences, Inc.

DECLARATION OF GENERATIVE AI AND AI-ASSISTED TECHNOLOGIES IN THE WRITING PROCESS

Generative AI was used to assist with copyediting of this manuscript. All authors reviewed and edited all generative AI suggestions and take full responsibility for this content.

STAR★METHODS

Detailed methods are provided in the online version of this paper and include the following:

- KEY RESOURCES TABLE
- METHOD DETAILS
  - Ultrasound transducer and drive system
  - Hydrophone scan measurements
  - Calibration model
  - Skull mounting
  - MRI scanning
  - MRI analysis
  - Acoustic simulations
  - Acoustic holography
- QUANTIFICATION AND STATISTICAL ANALYSIS

SUPPLEMENTAL INFORMATION

Supplemental information can be found online at https://doi.org/10.1016/j.crmeth.2026.101568.

Received: February 2, 2026
Revised: May 28, 2026
Accepted: July 23, 2026
Published: August 14, 2026

REFERENCES

1. Murphy, K., and Fouragnan, E. (2024). The future of transcranial ultrasound as a precision brain interface. PLoS Biol. *22*, e3002884. https://doi.org/10.1371/journal.pbio.3002884.
2. Fan, J.M., Woodworth, K., Murphy, K.R., Hinkley, L., Cohen, J.L., Yoshimura, J., Choi, I., Tremblay-McGaw, A.G., Mergenthaler, J., and Good, C.H. (2024). Thalamic transcranial ultrasound stimulation in treatment resistant depression. Brain Stimul. Basic Transl. Clin. Res. Neuromodulation. *17*, 1001–1004.
3. Attali, D., Tiennot, T., Manuel, T.J., Daniel, M., Houdouin, A., Annic, P., Dizeux, A., Haroche, A., Dadi, G., and Henensal, A. (2025). Deep transcranial ultrasound stimulation using personalized acoustic metamaterials improves treatment-resistant depression in humans. Brain Stimulat *18*, 1004–1014.
4. Subramaniam, K., Attalla, G., Mathew, M., Alvarez, J.L., Dadgar-Kiani, E., Mahadevan, R., Nagarajan, S., and Murphy, K.R. (2026). Low-intensity focused ultrasound targeting striatal circuits in schizophrenia: feasibility, safety, and effects on hallucinations and striatal-temporal functional connectivity. Brain Stimul *19*, 103084. https://doi.org/10.1016/j.brs.2026.103084.
5. Darmani, G., Bergmann, T.O., Butts Pauly, K., Caskey, C.F., de Lecea, L., Fomenko, A., Fouragnan, E., Legon, W., Murphy, K.R., Nandi, T., et al. (2022). Non-invasive transcranial ultrasound stimulation for neuromodulation. Clin. Neurophysiol. *135*, 51–73. https://doi.org/10.1016/j.clinph.2021.12.010.
6. Riis, T.S., Feldman, D.A., Losser, A.J., Okifuji, A., and Kubanek, J. (2024). Noninvasive targeted modulation of pain circuits with focused ultrasonic waves. Pain *165*, 2829–2839. https://doi.org/10.1097/j.pain.0000000000003322.
7. Fouragnan, E.F., Chau, B.K.H., Folloni, D., Kolling, N., Verhagen, L., Klein-Flügge, M., Tankelevitch, L., Papageorgiou, G.K., Aubry, J.F., Sallet, J., and Rushworth, M.F.S. (2019). The macaque anterior cingulate cortex translates counterfactual choice value into actual behavioral change. Nat. Neurosci. *22*, 797–808. https://doi.org/10.1038/s41593-019-0375-6.
8. Folloni, D., Verhagen, L., Mars, R.B., Fouragnan, E., Constans, C., Aubry, J.F., Rushworth, M.F.S., and Sallet, J. (2019). Manipulation of Subcortical and Deep Cortical Activity in the Primate Brain Using Transcranial Focused Ultrasound Stimulation. Neuron *101*, 1109–1116.e5. https://doi.org/10.1016/j.neuron.2019.01.019.
9. Priestley, L., Chiew, M., Shahdloo, M., Mahmoodi, A., Cheng, X., Cleveland, R., Rushworth, M., and Khalighinejad, N. (2024). Dorsal raphe nucleus controls motivational state transitions in monkeys. Preprint at bioRxiv. https://doi.org/10.1101/2024.02.13.580224.
10. Martin, E., Roberts, M., Grigoras, I.F., Wright, O., Nandi, T., Rieger, S.W., Campbell, J., den Boer, T., Cox, B.T., Stagg, C.J., and Treeby, B.E. (2025). Ultrasound system for precise neuromodulation of human deep brain circuits. Nat. Commun. *16*, 8024. https://doi.org/10.1038/s41467-025-63020-1.
11. Murphy, K.R., Farrell, J.S., Bendig, J., Mitra, A., Luff, C., Stelzer, I.A., Yamaguchi, H., Angelakos, C.C., Choi, M., Bian, W., et al. (2024). Optimized ultrasound neuromodulation for non-invasive control of behavior and physiology. Neuron *112*, 3252–3266.e5. https://doi.org/10.1016/j.neuron.2024.07.002.
12. Murphy, K.R., Nandi, T., Kop, B., Osada, T., Lueckel, M., N'Djin, W.A., Caulfield, K.A., Fomenko, A., Siebner, H.R., Ugawa, Y., et al. (2025). A practical guide to transcranial ultrasonic stimulation from the IFCN-endorsed ITRUSST consortium. Clin. Neurophysiol. *171*, 192–226. https://doi.org/10.1016/j.clinph.2025.01.004.
13. Martin, E., Aubry, J.-F., Schafer, M., Verhagen, L., Treeby, B., and Pauly, K.B. (2024). ITRUSST consensus on standardised reporting for transcranial ultrasound stimulation. Brain Stimulat *17*, 607–615.
14. Aubry, J.-F., Attali, D., Schafer, M.E., Fouragnan, E., Caskey, C.F., Chen, R., Darmani, G., Bubrick, E.J., Sallet, J., and Butler, C.R. (2025). ITRUSST consensus on biophysical safety for transcranial ultrasound stimulation. Brain Stimulat *18*, 1896–1905.
15. Kreider, W., Yuldashev, P.V., Sapozhnikov, O.A., Farr, N., Partanen, A., Bailey, M.R., and Khokhlova, V.A. (2013). Characterization of a multi-element clinical HIFU system using acoustic holography and nonlinear modeling. IEEE Trans. Ultrason. Ferroelectr. Freq. Control *60*, 1683–1698. https://doi.org/10.1109/TUFFC.2013.2750.
16. Sapozhnikov, O.A., Tsysar, S.A., Khokhlova, V.A., and Kreider, W. (2015). Acoustic holography as a metrological tool for characterizing medical ultrasound sources and fields. J. Acoust. Soc. Am. *138*, 1515–1532. https://doi.org/10.1121/1.4928396.
17. Martin, E., Roberts, M., and Treeby, B. (2021). Measurement and simulation of steered acoustic fields generated by a multielement array for therapeutic ultrasound. JASA Express Lett. *1*, 012001. https://doi.org/10.1121/10.0003210.
18. Williams, R.P., Kreider, W., Nartov, F.A., Karzova, M.M., Khokhlova, V.A., Sapozhnikov, O.A., and Khokhlova, T.D. (2025). Synthesized acoustic holography: A method to evaluate steering and focusing performance of ultrasound arrays. J. Acoust. Soc. Am. *157*, 2750–2762. https://doi.org/10.1121/10.0036225.
19. Ghanem, M.A., Maxwell, A.D., Kreider, W., Cunitz, B.W., Khokhlova, V.A., Sapozhnikov, O.A., and Bailey, M.R. (2018). Field characterization and compensation of vibrational nonuniformity for a 256-element focused ultrasound phased array. IEEE Trans. Ultrason. Ferroelectr. Freq. Control *65*, 1618–1630. https://doi.org/10.1109/TUFFC.2018.2851188.
20. Pinton, G., Aubry, J.F., Bossy, E., Muller, M., Pernot, M., and Tanter, M. (2012). Attenuation, scattering, and absorption of ultrasound in the skull bone. Med. Phys. *39*, 299–307. https://doi.org/10.1118/1.3668316.
21. Treeby, B.E., and Cox, B.T. (2010). k-Wave: MATLAB toolbox for the simulation and reconstruction of photoacoustic wave fields. J Biomed Opt *15*, 021314. https://doi.org/10.1117/1.3360308.
22. Pichardo, S. (2023). BabelBrain: An Open-Source Application for Prospective Modeling of Transcranial Focused Ultrasound for Neuromodulation Applications. IEEE Trans. Ultrason. Ferroelectr. Freq. Control *70*, 587–599.
23. Miscouridou, M., Pineda-Pardo, J.A., Stagg, C.J., Treeby, B.E., and Stanziola, A. (2022). Classical and Learned MR to Pseudo-CT Mappings for Accurate Transcranial Ultrasound Simulation. IEEE Trans. Ultrason. Ferroelectr. Freq. Control *69*, 2896–2905. https://doi.org/10.1109/TUFFC.2022.3198522.
24. Attali, D., Tiennot, T., Schafer, M., Fouragnan, E., Sallet, J., Caskey, C.F., Chen, R., Darmani, G., Bubrick, E.J., Butler, C., et al. (2023). Three-layer model with absorption for conservative estimation of the maximum acoustic transmission coefficient through the human skull for transcranial ultrasound stimulation. Brain Stimul. *16*, 48–55. https://doi.org/10.1016/j.brs.2022.12.005.
25. Gordon, C.C., Blackwell, C.L., Bradtmiller, B., Parham, J.L., Barrientos, P., Paquette, S.P., Corner, B.D., Carson, J.M., Venezia, J.C., Rockwell, B.M., et al. (2014). 2012 Anthropometric Survey of U.S. Army Personnel: Methods and Summary Statistics. Security. https://api.semanticscholar.org/CorpusID:107330720
26. Souza, R., Lucena, O., Garrafa, J., Gobbi, D., Saluzzi, M., Appenzeller, S., Rittner, L., Frayne, R., and Lotufo, R. (2018). An open, multi-vendor, multi-field-strength brain MR dataset and analysis of publicly available skull stripping methods agreement. Neuroimage *170*, 482–494. https://doi.org/10.1016/j.neuroimage.2017.08.021.
27. Eskildsen, S.F., Coupé, P., Fonov, V., Manjón, J.V., Leung, K.K., Guizard, N., Wassef, S.N., Østergaard, L.R., and Collins, D.L.; Alzheimer's Disease Neuroimaging Initiative (2012). BEaST: brain extraction based on nonlocal segmentation technique. Neuroimage *59*, 2362–2373. https://doi.org/10.1016/j.neuroimage.2011.09.012.
28. Ledig, C., Schuh, A., Guerrero, R., Heckemann, R.A., and Rueckert, D. (2018). Structural brain imaging in Alzheimer's disease and mild cognitive

CellPress
OPEN ACCESS

impairment: biomarker analysis and shared morphometry database. Sci. Rep. *8*, 11258. https://doi.org/10.1038/s41598-018-29295-9.

29. Ilham, S.J., and Kiani, M. (2023). Towards high-resolution ultrasound neuromodulation with crossed-beam phased arrays. IEEE Trans. Biomed. Circuits Syst. *17*, 534–546. https://doi.org/10.1109/TBCAS.2023.3285724.

30. Riis, T., Feldman, D., Mickey, B., and Kubanek, J. (2024). Controlled noninvasive modulation of deep brain regions in humans. Commun. Eng. *3*, 13. https://doi.org/10.1038/s44172-023-00146-4.

31. Virtanen, P., Gommers, R., Oliphant, T.E., Haberland, M., Reddy, T., Cournapeau, D., Burovski, E., Peterson, P., Weckesser, W., Bright, J., et al. (2020). SciPy 1.0: fundamental algorithms for scientific computing in Python. Nat. Methods *17*, 261–272. https://doi.org/10.1038/s41592-019-0686-2.

32. Satopaa, V., Albrecht, J., Irwin, D., and Raghavan, B. (2011). Finding a "kneedle" in a haystack: Detecting knee Points in System Behavior (IEEE), pp. 166–171.

33. Çiçek, Ö., Abdulkadir, A., Lienkamp, S.S., Brox, T., and Ronneberger, O. (2016). 3D U-Net: Learning Dense Volumetric Segmentation from Sparse Annotation (Springer), pp. 424–432.

34. Caulfield, K.A., Sege, C.T., Weaver, J., Murphy, K.R., Passaro, T., Meads, K., Dadgar-Kiani, E., Antonucci, M.U., Good, C., and McTeague, L.M. (2026). Focused ultrasound to bilateral thalamic nuclei significantly improves human cognitive attention in a frequency- and intensity-dependent manner. Brain Stimul *19*, 103171. https://doi.org/10.1016/j.brs.2026.103171.

35. Miscouridou, M., Krokhmal, A.A., Hess, A.T., Campbell, J., Stagg, C.J., Martin, E., and Treeby, B.E. (2025). Pointwise Encoding Time reduction with Radial Acquisition (PETRA) to pseudo-CT mapping for precision Focused Transcranial Ultrasound Applications. Preprint at arXiv. https://doi.org/10.48550/arXiv.2508.01050.

36. Leung, S.A., Webb, T.D., Bitton, R.R., Ghanouni, P., and Butts Pauly, K. (2019). A rapid beam simulation framework for transcranial focused ultrasound. Sci. Rep. *9*, 7965. https://doi.org/10.1038/s41598-019-43775-6.

STAR★METHODS

KEY RESOURCES TABLE

REAGENT or RESOURCE	SOURCE	IDENTIFIER
Deposited data		
Measured and simulated focal pressures	This paper; Zenodo	https://doi.org/10.5281/zenodo.20840906
Biological samples		
Research Human Skull	Skulls Unlimited International, Inc.	https://www.skullsunlimited.com/pages/skulls-unlimiteds-research-skulls-and-skeletons
Software and algorithms		
MATLAB (R2024a)	The MathWorks Inc.	https://www.mathworks.com/products/matlab.html
k-Wave (v1.4.0)	Treeby et al.[21]	http://k-wave.org/
SciPy (v1.14.1)	Virtanen et al.[31]	https://github.com/scipy/scipy
Kneed (v0.8.5)	Satopaa et al.[32]	https://github.com/arvkevi/kneed
FUS safety	This paper	https://github.com/dadgarki/fus_safety_paper

METHOD DETAILS

Ultrasound transducer and drive system

A custom sparse 2D array containing 64 elements (Figure S2A) was manufactured (Sonic Concepts Inc.) and driven at 500 kHz by a custom drive system, ATTN201 (Attune Neurosciences Inc.). Each array element was rectangular (4.15 mm × 2.7 mm). When performing scan tank measurements, the array was pulsed at 70 V (peak-to-peak), a 5 Hz pulse repetition frequency, and a duty cycle of 1%.

For bowl-shaped ultrasound transducer measurements (Figure 2A), a high-power matrix array transducer (IMASONIC, Voray-sur-l'Ognon, France) was used, consisting of 128 transmitting elements arranged in a Fermat spiral and a transmit frequency of 900 kHz.

When simulating variant ultrasound array designs (Figure S2A), a concentric circle layout with 3.8 mm diameter circular elements, a Fermat spiral layout with 3.8 mm diameter circular elements, and a grid with 3.38 mm square elements were used. All variant designs contained 64 elements.

Hydrophone scan measurements

Hydrophone scan tank measurements were made using a capsule hydrophone (ONDA HGL-400) calibrated at 500 kHz mounted in the ONDA AIMS III scan tank (Product # AST3-L-3). Directivity loss compensation was calculated using a Bessel function fit as specified by the manufacturer. Water was degassed and filtered continued with an Aquas-10 Water Conditioning system (Onda product # AQUAS-10-110V).

When steering the ultrasound transducer to a given target point, the hydrophone and scan tank system were used to measure the peak pressure at each point in an XY plane (0.5 mm resolution) at the target point and orthogonal to the transducer array. The peak pressure value in this plane was used as the focal pressure. Directivity correction was performed to each pressure measurement using the hydrophone's provided Bessel function, which considers the angle between the transducer and hydrophone to yield a pressure correction factor.

To calibrate the separate bowl-shaped transducer, the geometric focus of the transducer was first found by completing a 3D pressure mapping around the expected geometric focus using a 3D motor stage (Image Guided Therapy, Pessac France) and an HNC-0400 hydrophone (Onda, Sunnyvale CA). Its focus was then steered in a 6 × 6 × 6 grid with a 2D pressure map (0.5 mm resolution) collected for each focal point. The grid spanned from −15 mm from the geometric focus to 15 mm from the geometric focus in all dimensions. Simulations were then completed in MATLAB using k-Wave at all locations.

Calibration model

To fit the calibration model to the measured spatial discrepancy between measured and simulated peak pressures, we optimized the following generalized Gaussian function:

$$\Delta P(\vec{x}) = a\, e^{-\left(\vec{x}-\vec{x}_0\right)^T B \left(\vec{x}-\vec{x}_0\right)} + c$$

The scalar constants a, c, and diagonal matrix B were set by minimizing the mean squared error between measured and simulated peak pressures for all target points and skull fragments. For 2D spatial calibrations (Figures 1D–1F), B was represented as a 2×2 diagonal matrix, whereas for the 3D spatial calibrations (Figures 1G–1K), B was represented as a 3×3 diagonal matrix. Other model fits were performed were: planar ($A\vec{x} + b$), radial decay ($a\,e^{-b\vec{x}^T\vec{x}} + c$), quadratic surface ($ax^2+by^2+cz^2+dxy+exz+fyz+gx+hy+kz+l$), isotropic Gaussian ($a\,e^{-b\left(\vec{x}-\vec{x}_0\right)^T\left(\vec{x}-\vec{x}_0\right)} + c$), and stepwise elliptical:

$$f(x) = \begin{cases} d, \text{if } (x - x_0)^2 a + (y - y_0)^2 b + (z - z_0)^2 c < 1 \\ 0, \textit{otherwise} \end{cases} + e$$

Prior to model fit, all variables were standardized by subtracting their mean and dividing by their standard deviation. Non-linear least squares model fit was performed using the Levenberg-Marquardt algorithm through the scipy.optimize.curve_fit function available in the Python SciPy package,[31] with model parameters containing an initial value of 1, except for constant offsets which were set to 0.

For determining the requisite number of sample points for a model fit, the relationship between number of sample points and R^2 was first found through random subsampling. The mean value of this function was passed through an automatic elbow detection algorithm called the Kneedle algorithm,[32] through the Python package *kneed*. The algorithm locates the point of maximum curvature, corresponding to the number of sample points at which additional samples provide diminishing returns.

Skull mounting

Cadaver skulls were obtained from Skulls Unlimited International, Inc. Transducer positioning was set by placing an ATTN201 headset on the skull with 5 mm foam padding at the skull intersection points to mimic scalp thickness offset. The distance between each fiducial and the orthogonal skull intercept was measured by first inserting a thin metal rod through $^1/_8$ inch drilled holes in each fiducial until it touched the skull. A 3D printed washer was fitted tightly to the rod and pressed flush against the outer surface of the transducer while inserting the rod to ensure orthogonal alignment. To mark the length of the measurement rod from the fiducial to the skull, the rod and tightly fit washer were removed from the headset placed on the skull and reinserted to the corresponding fiducial on an identical headset until the washer was flush against the outer surface. A felt marker was then used to mark the distance from the tip of the rod to the inner transducer surface, then measured with calipers once the rod was removed. The intersect position on the skull was then marked by dabbing the measurement rod in red acrylic paint and re-inserting the rod into the headset on the skull until the washer was flush with the outer transducer surface and the rod was therefore touching the skull. This process was repeated for each fiducial.

To secure the bracket to the skull at the correct distance and angle from each fiducial, conical shaped spacers the length of the previously measured distances between the fiducials and orthogonal skull were 3D printed and super glued to a fiducial puck at each location (Figure 3A). A sharpie was used to mark the center of the fiducial and assist in aligning the spacer with the center of each fiducial. This puck was then placed in the bracket and the fiducial spacers were aligned with the corresponding paint marks on the skull. To secure the bracket to the skull, holes were first drilled into the bracket. Clay was fit around the skull to allow for orthogonal drilling through the bracket in 3 locations. The skull segment surrounding the bracket was traced then extracted with a drill. Finally, the bracket was secured to the skull fragment by aligning the fiducial puck with the paint marks on the skull segment once again then adding screws to hold the bracket. Glue was added sparingly to secure the screws in place then the fiducial puck was removed. Prior to experiments, skulls were degassed in a plexiglass chamber filled with deionized water using a vacuum pump at −30 Hg for at least 48 h. In total, 10 skull fragments from 5 cadaver skulls were obtained. Two fragments and their corresponding MRI scans were not used for acoustic simulations due to damaged bone.

MRI scanning

Whole head scanning was performed on a Siemens MAGNETOM Cima.X 3T scanner with a 64-channel head coil. MPRAGE (0.94 mm isotropic resolution) and ZTE (0.75 mm isotropic resolution) scans were collected for each of 157 subjects while simultaneously wearing an MRI-compatible version of the mock headset, using key scan parameters derived previously.[23] ZTE scans were also acquired for 5 mounted *ex vivo* skull fragments with MRI fiducials while submerged in a plastic bag containing previously degassed filtered water.

MRI analysis

Spherical fiducial markers (8 total; 4 on each side) in the headset were visible with high MR contrast and used to align the MPRAGE and ZTE scans to a common coordinate system. Brains were extracted from the MPRAGE volumes using a custom 3D U-Net model[33] trained on a large open source dataset.[26–28] Brains were then aligned with both the Allen and MNI reference atlases using the ANTs software package to derive target coordinates corresponding to several deep brain regions. The deep brain regions (Centromedian nucleus of the thalamus, Caudate, Amygdala, and Subthalamic Nucleus) were selected based on published and ongoing studies using our custom ultrasound device.[2,4,34] Skull masks were automatically extracted from ZTE scans using another custom 3D U-Net model[33] trained on a subset of manually annotated data.

Acoustic simulations

Acoustic simulations were performed in MATLAB R2024a using the k-Wave v1.4.0 toolbox and kspaceFirstOrder3D function.[21] Simulation parameters include a Courant-Friedrichs-Lewy (CFL) value of 0.3, BonA nonlinearity parameter value of 6, power law

absorption exponent of 1.5, isotropic spatial sampling of 0.7 mm for the sparse 2D transducer or 0.2 mm for the bowl transducer, and 20 cycle sinusoidal pulsing to reach pseudo-steady state. Peak simulation values were captured from the entire simulation time domain. All simulations for the sparse 2D transducer were performed on a desktop running Ubuntu 24.04.2 LTS with an NVIDIA GeForce RTX 4070 GPU. Simulations of the bowl transducer were performed on a Quadro P6000 GPU.

Five rigidly aligned CT and ZTE bone fragment scans (Figure S4A) were used to derive a linear relationship between Hounsfield units and ZTE voxel units. A robust principal component analysis of corresponding CT and ZTE voxel values yielded the following equation based on the primary principal component, $CT = -3609.8 \times ZTE + 3258.8$ (Figure 3B). This fit generally agrees with that from Miscouridou et al.,[35] $CT = -2929.6 \times ZTE + 3274.9$, as confirmed through direct comparisons of resulting focal pressure, which showed near parity (Figure S4D). While direct conversion for each fragment would have given idealized skull properties, as it was important to base our analysis on generalizable, real-world use cases, we only used this data to validate the existing ZTE-CT mapping provided in literature.[23] Using the mapping from Miscouridou et al.,[35] voxel-level ZTE values were converted to a Pseudo-CT before being used to calculate speed of sound and density values for use in the acoustic wave propagation equations.

To steer through a bone fragment and maximize pressure at a given coordinate, a time reversal simulation was used to calculate the individual element phases to be used in a subsequent forward simulation. For obtaining a globally optimal bone attenuation coefficient across the 8 *ex vivo* temporal window bone fragments, the attenuation coefficient was first swept through a range of values from 0 to 40. For a given alpha value, we simulated the propagation of acoustic waves from transducer elements through each *ex vivo* bone fragment and targeting each of the forty projected coordinates (Figure 3D). The globally optimal bone attenuation coefficient was selected that minimized the mean squared error between simulated target pressure and measured target pressure across all bone fragments and target positions.

Thermal simulations were performed using the Pennes' bioheat equation, available through the kWaveDiffusion function in the k-Wave v1.4.0 toolbox. The fraction of attenuation transformed to heat in bone was set to 0.16 based on previous studies. Specific heat (3630 J/kg/K), thermal conductivity (0.51 W/m/K), blood density (1050 kg/m^3), blood specific heat (3617 J/kg/K), blood perfusion rate (0.01 L/s), blood ambient temperature (37°C), and initial temperature distribution (37°C) were all based on previous studies.[36]

Acoustic holography

Acoustic holography was used to calibrate a custom sparse 2D phased array transducer by measuring the complex acoustic field generated by each element over a two-dimensional plane. A capsule hydrophone (ONDA HGL-400), calibrated at 500 kHz and mounted in an ONDA AIMS III scan tank (AST3-L-3), was raster-scanned at a fixed axial depth of 30 mm across a 120 mm × 120 mm region using a uniform spatial step size of 0.7 mm. At each (x, y) position, individual array elements were driven sequentially by a Verasonics Vantage NXT system, which emitted a 500-kHz, 100-μs pulse for each element followed by a 100-μs inter-element delay. For every transmitted pulse, the resulting hydrophone waveform was recorded using a PicoScope PS5000A oscilloscope.

Recorded signals were converted from ADC units to millivolts and processed offline. Waveforms were bandpass-filtered (400–500 kHz) using a zero-phase Butterworth filter, and the analytic signal was computed via the Hilbert transform to extract instantaneous amplitude and phase. Because elements were fired with fixed timing, each element's contribution appeared in a known temporal window. For each element at each spatial location, phase was sampled at a fixed time corresponding to the onset of the pulse, and amplitude was quantified as the peak negative pressure within a defined analysis window, using the hydrophone sensitivity of 6.2 V/MPa. These measurements were compiled into three-dimensional arrays indexed by lateral position, elevational position, and element number, yielding a complete holographic map of the element-wise acoustic field for calibration and field reconstruction.

For each element's complex hologram, angular spectrum backpropagation was performed using the angularSpectrumCW function in the k-Wave v1.4.0 toolbox in order to recover the phase and amplitude in the source plane. For use in focusing in a forward propagation simulation, each complex source plane could be phase offset based on the element's distance to the target, and all planes could then be summed together. For forward propagation, the same angularSpectrumCW function was used.

QUANTIFICATION AND STATISTICAL ANALYSIS

This study reports descriptive statistics and uncertainty estimate bounds, rather than null-hypothesis significance tests. Focal calibration model fits were assessed using the coefficient of determination (R^2), and model comparison among candidate functions was performed using Bayesian model selection (Figures 1K and 2D). An automatic elbow detection algorithm[32] was applied to the R^2-vs-sample-size curve to determine the minimum sample count needed for a stable fit. For the *ex vivo* skull fragments, the 5th and 95th percentile values (Figure 3F) were directly obtained from the distribution of percent pressure discrepancies, defined as (measured – simulated)/simulated. For worst-case thermal analysis, the attenuation coefficient lower and upper bounds were selected by computing the mean coefficient with percent pressure discrepancies closest to the 5th and 95th percentiles (Figure S5B). Group-level summary statistics are reported as mean ± standard deviation. Number of independent samples in each analysis is specified in relevant figure legends (N = 157 subjects for *in vivo* anatomy and virtual temporal windows, N = 8 fragments for attenuation coefficient optimization, and N = 5 fragments for validation of PETRA to CT mapping). All quantification and statistical analyses were performed in Python and MATLAB.

Cell Reports Methods, Volume *6*

Supplemental information

Calibrated simulations for dynamic focusing of ultrasound through the temporal window

Ehsan Dadgar-Kiani, Vibha Hebbale, Malachy W. Newman, Grace Attalla, John L. Alvarez, Suraya Dunsford, Kevin A. Caulfield, Cameron H. Good, Andrew D. Krystal, Leo P. Sugrue, Joline M. Fan, Elsa Fouragnan, Charles F. Caskey, Samuel Pichardo, Kim Butts Pauly, and Keith R. Murphy

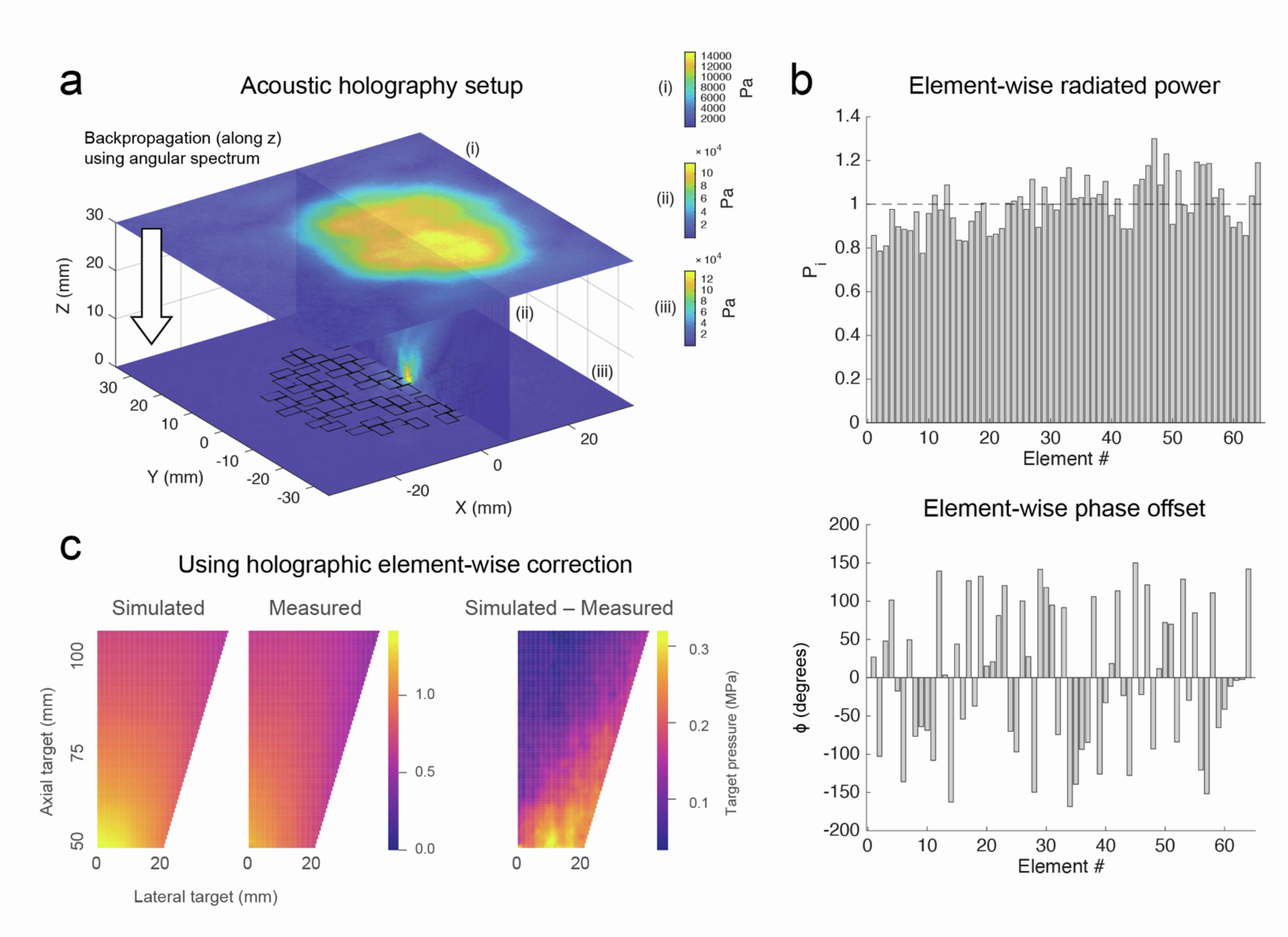

Figure S1. Holography-based transducer calibration is unable to capture spatially dependent crosstalk when steering a multi-element phased array, Related to Figure 1. **a**) Depiction of the acoustic holography setup used to calibrate a 2D phased array transducer by measuring the complex acoustic field generated by each element over a two-dimensional plane at a distance of 30 mm from the array plane. This measured complex plane (i) from a single source element is backpropagated using the angular spectrum method to recover the source complex plane (iii). An orthogonal plane (ii) depicts the spatial convergence of the pressure field to the original source element through backpropagation. In (i), (ii), and (iii), only complex magnitude is depicted. **b**) Following backpropagation for each source element, variability in average radiated power and phase offset are observed for each element. **c**) When using the source complex planes to simulate focusing across the steering range, the discrepancy between simulated peak pressures and measured peak pressures still shows a significant spatial dependence, indicating that purely holographic-based transducer calibration is insufficient.

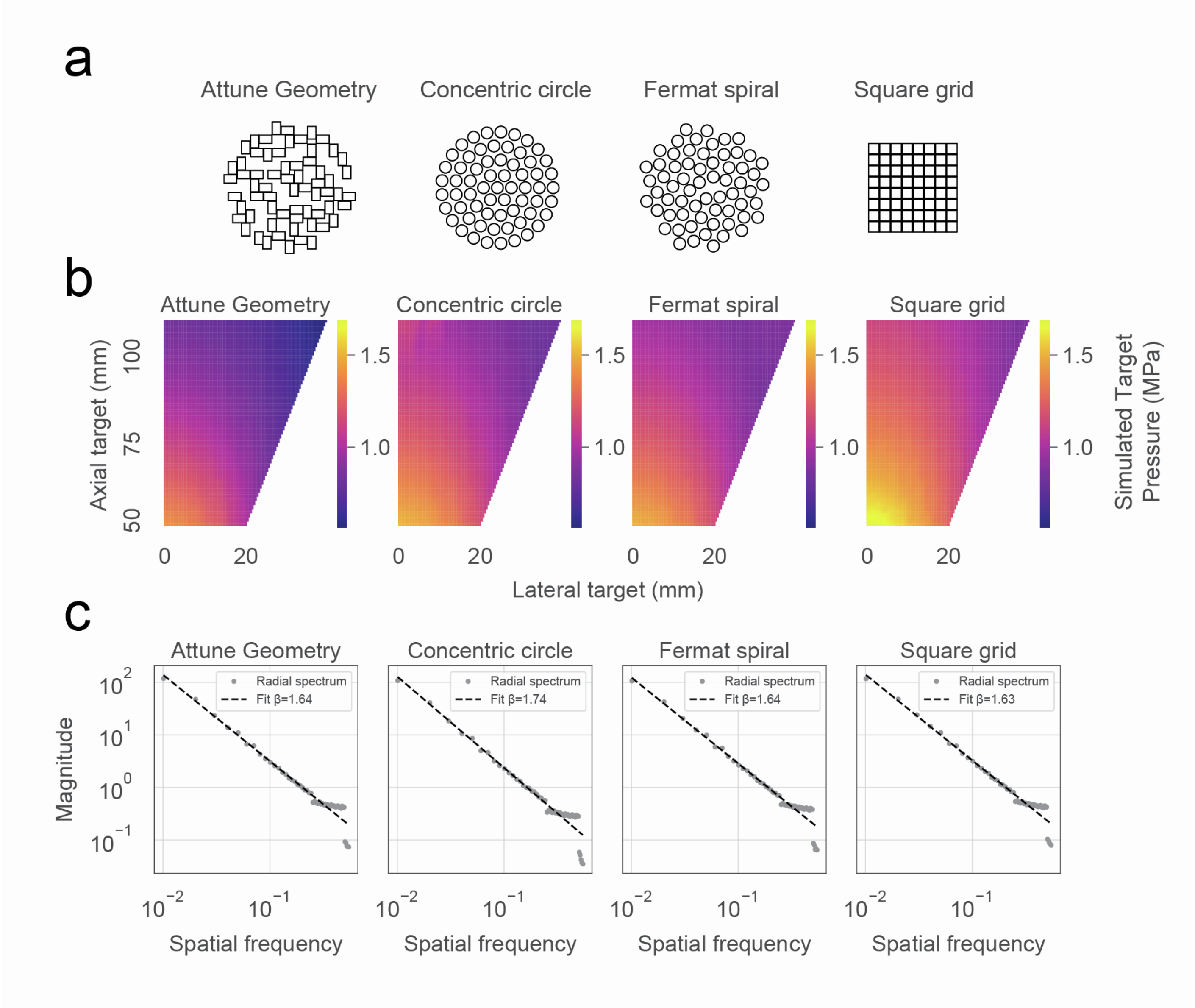

Figure S2. Variant ultrasound array designs tested for focal pressure output symmetry, Related to Figure 1. **a**) Depictions of 2D transducer element configurations used to generate **b**) simulated focal pressure maps when steering across a 2D calibration range. **c**) All array configurations' focal pressure maps showed comparable magnitudes across the relevant spatial frequency range.

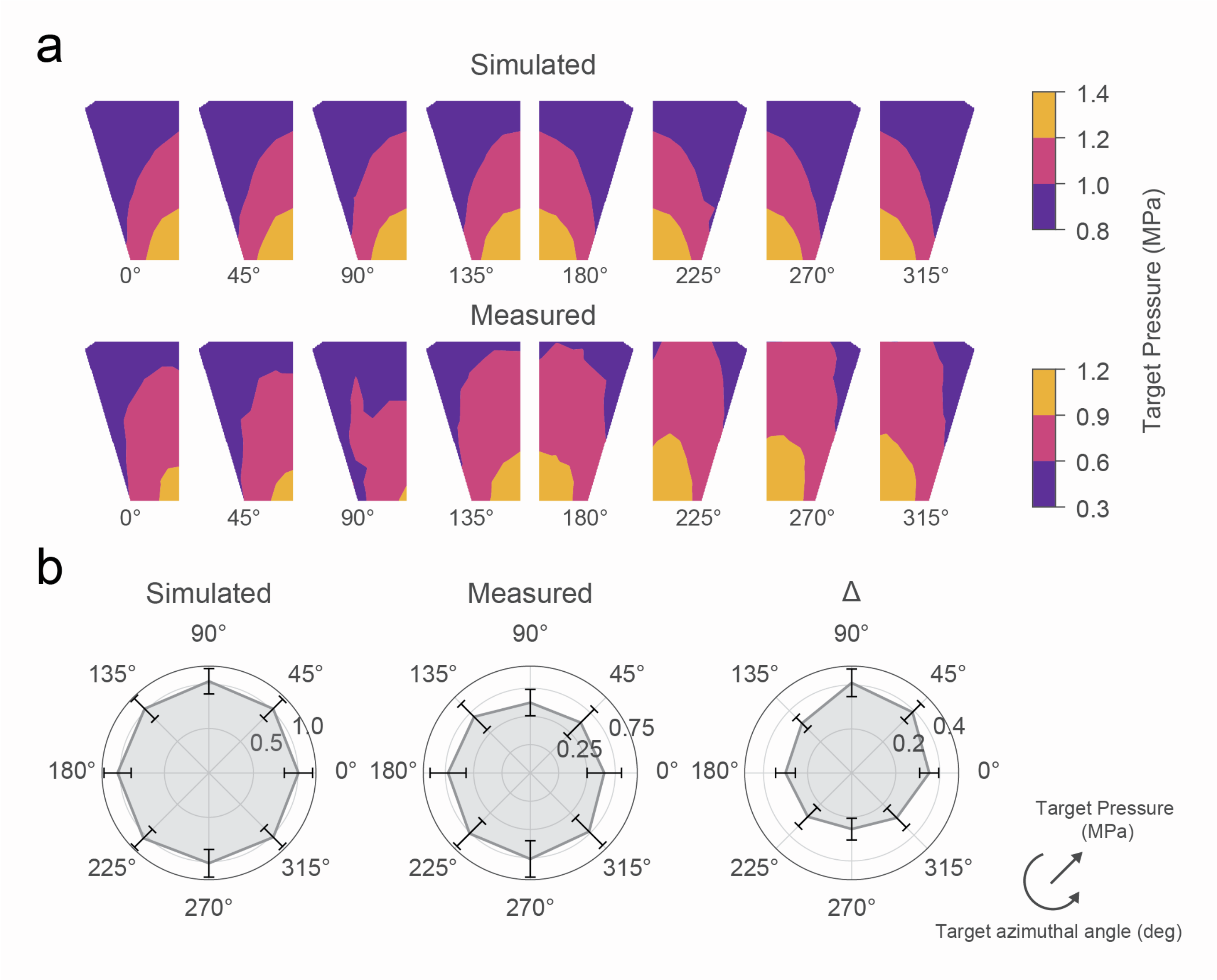

Figure S3. Focal pressures at variable azimuthal angles show spatial nonuniformity and lateral bias between measurement and simulation, Related to Figure 1. a) Comparisons between simulated and measured focal pressure cross sections ranging from 0º to 315º in 45º increments. Maps were segmented into three uniform color bins to better depict the uniformity evident in simulation but absent in measurement. **b)** Radar charts of the focal pressures and discrepancies between measured and simulated values shows a lateral bias towards 45º and 90º that would not be captured in uncorrected simulations. Results are represented as mean ± SD.

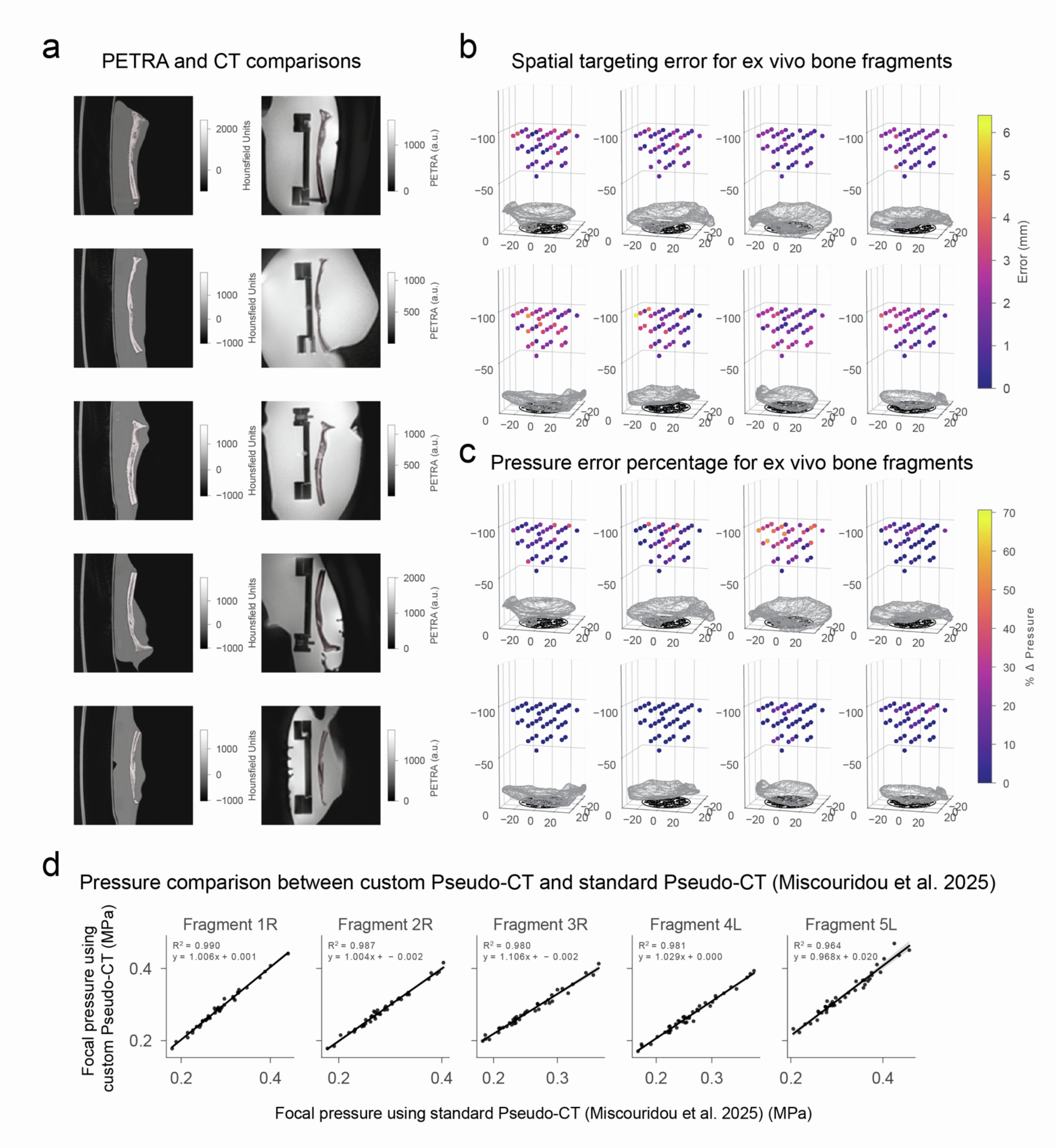

Figure S4. Spatial targeting errors and simulated focal pressure errors through mounted temporal window skull fragments, Related to Figure 3. **a**) Comparisons of CT and ZTE scans acquired for five fragments after rigid alignment between their bone masks. **b**) Depictions of each fragment with targets used for optimizing the bone alpha coefficient. Target points are color-coded by spatial error (mm) between intended and measured focal peak in the target plane. **c**) Similar depictions of the percentage error between measured and simulated target pressure at each point when using the optimized attenuation coefficient (15.3 dB/cm/MHz). **d**) For each of the five fragments, comparisons between the focal pressures from separately using the standard

Pseudo-CT fit (Miscouridou et al.[1]) or the custom Pseudo-CT fit confirms a highly linear relationship between the two mapping methods.

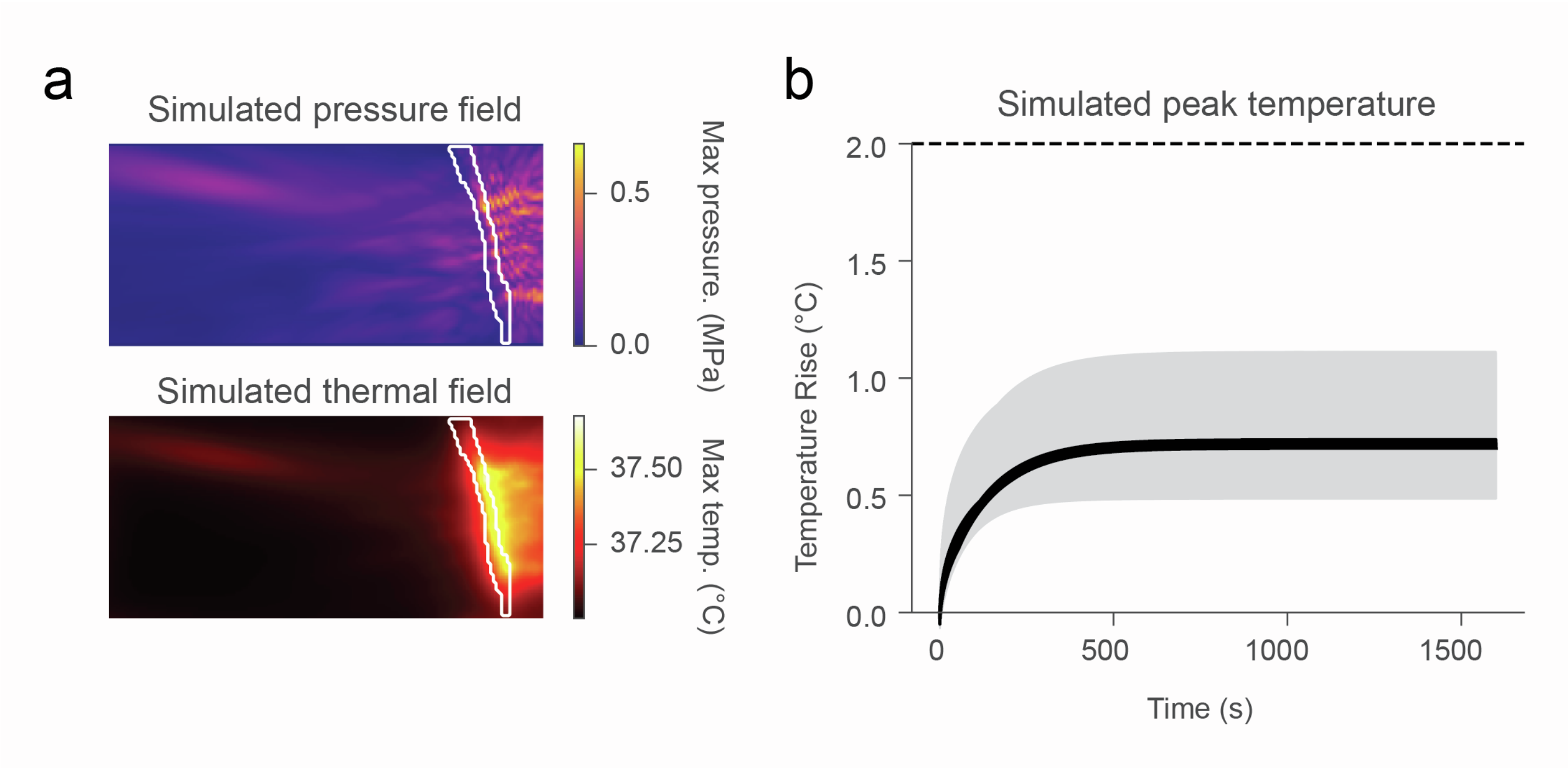

Figure S5. Safety considerations when simulating worst-case temporal window temperature rise, Related to Figure 3. **a**) Cross section of simulated pressure and thermal fields depicting max pressure and max temperature, respectively. White contours indicate the fragment bone mask. **b**) Simulated dynamics of maximum temperature rise in a bone fragment over the course of a 30-minute Pennes' bioheat simulation with bone attenuation coefficient of 15.3 dB/cm/MHz. Shaded band indicates range of peak temperatures when considering worst- and best-case attenuations in the range of 3.1 to 31.0 dB/cm/MHz.

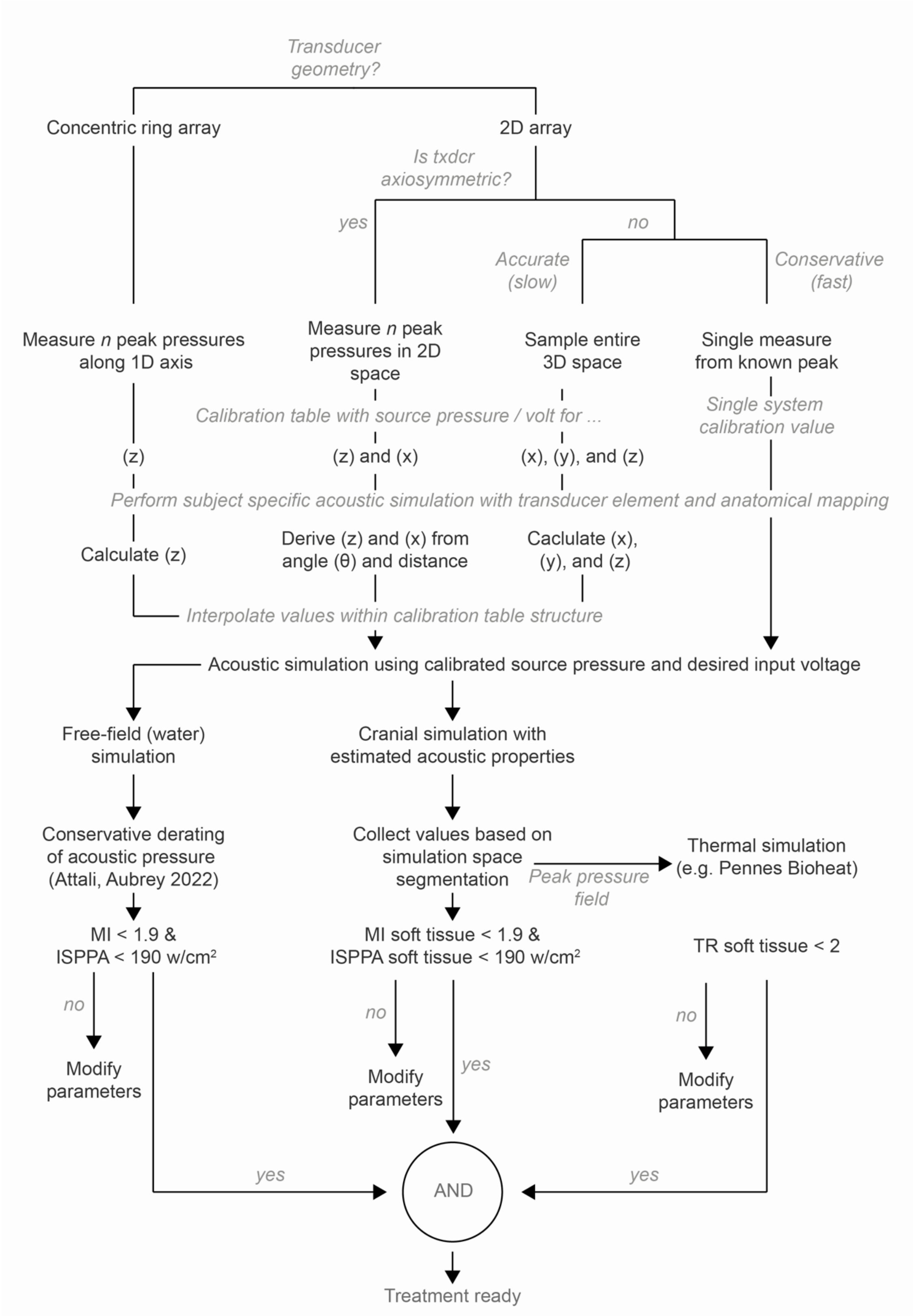

Figure S6. Algorithmic approach to array calibration, Related to STAR Methods.

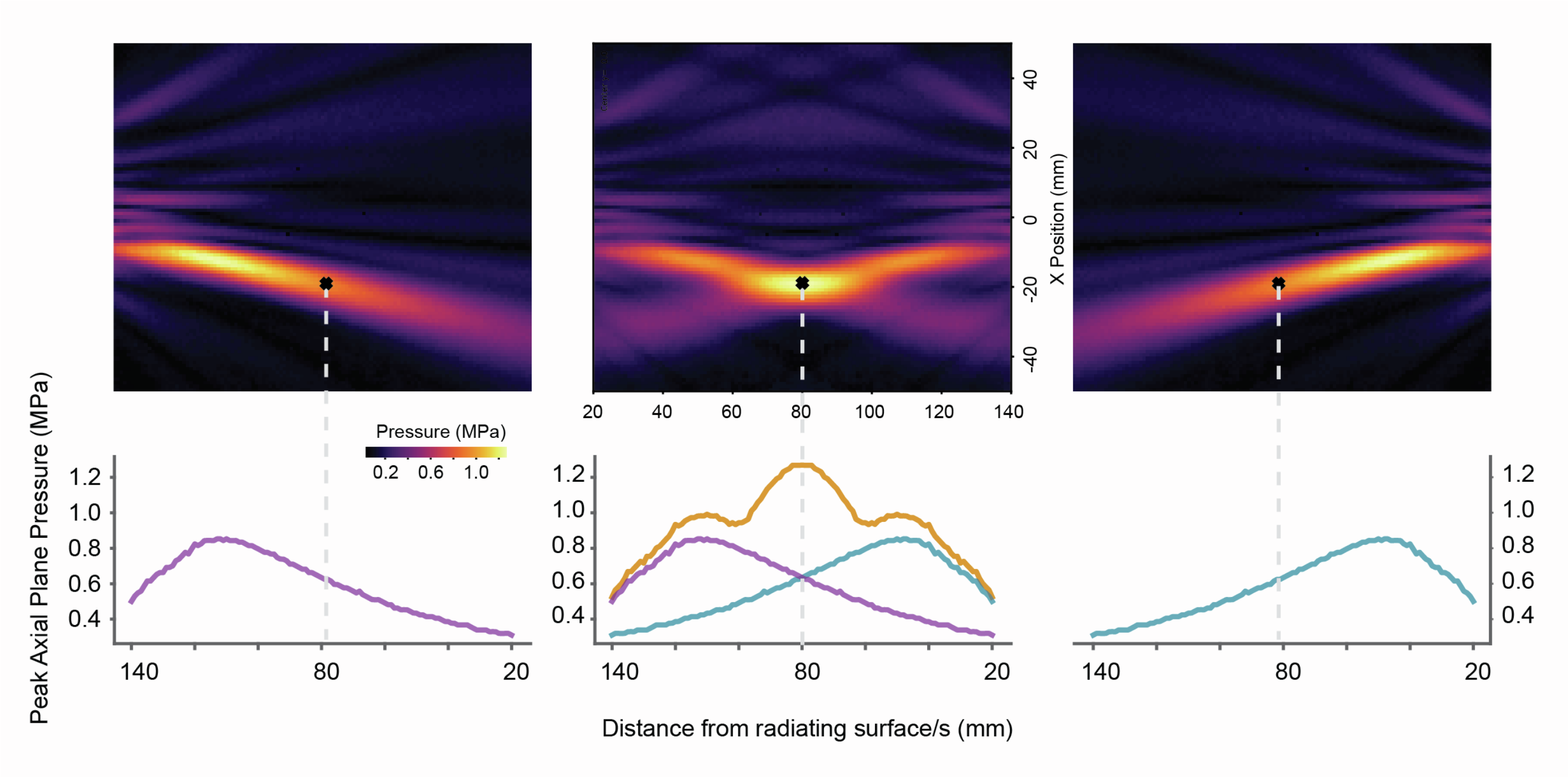

Figure S7. Single array focusing may result in near-field shifted peak pressure, Related to STAR Methods. Intersectional cross beams (Left, Right) where the intended focal positions are summed exceeds the peak pressure of independent near field pressures (Center).

REFERENCES

1. Miscouridou, M., Krokhmal, A.A., Hess, A.T., Campbell, J., Stagg, C.J., Martin, E., and Treeby, B.E. (2025). Pointwise Encoding Time reduction with Radial Acquisition (PETRA) to pseudo-CT mapping for precision Focused Transcranial Ultrasound Applications. Preprint at arXiv, https://doi.org/10.48550/arXiv.2508.01050 https://doi.org/10.48550/arXiv.2508.01050.
